# A new microvertebrate assemblage from the Mussentuchit Member, Cedar Mountain Formation: insights into the paleobiodiversity and paleobiogeography of early Late Cretaceous ecosystems in western North America

**DOI:** 10.7717/peerj.5883

**Published:** 2018-11-16

**Authors:** Haviv M. Avrahami, Terry A. Gates, Andrew B. Heckert, Peter J. Makovicky, Lindsay E. Zanno

**Affiliations:** 1 Department of Biological Sciences, North Carolina State University, Raleigh, NC, USA; 2 North Carolina Museum of Natural Sciences, Raleigh, NC, USA; 3 Department of Geological and Environmental Sciences, Appalachian State University, Boone, NC, USA; 4 Field Museum of Natural History, Chicago, IL, USA

**Keywords:** Biodiversity, Upper Cretaceous, Cedar mountain formation, Cenomanian, Microvertebrate, Dinosaur, Vertebrate, Mussentuchit Member

## Abstract

The vertebrate fauna of the Late Cretaceous Mussentuchit Member of the Cedar Mountain Formation has been studied for nearly three decades, yet the fossil-rich unit continues to produce new information about life in western North America approximately 97 million years ago. Here we report on the composition of the Cliffs of Insanity (COI) microvertebrate locality, a newly sampled site containing perhaps one of the densest concentrations of microvertebrate fossils yet discovered in the Mussentuchit Member. The COI locality preserves osteichthyan, lissamphibian, testudinatan, mesoeucrocodylian, dinosaurian, metatherian, and trace fossil remains and is among the most taxonomically rich microvertebrate localities in the Mussentuchit Member. To better refine taxonomic identifications of isolated theropod dinosaur teeth, we used quantitative analyses of taxonomically comprehensive databases of theropod tooth measurements, adding new data on theropod tooth morphodiversity in this poorly understood interval. We further provide the first descriptions of tyrannosauroid premaxillary teeth and document the earliest North American record of adocid remains, extending the appearance of this ancestrally Asian clade by 5 million years in western North America and supporting studies of pre-Cenomaninan Laurasian faunal exchange across Beringia. The overabundance of mesoeucrocodylian remains at the COI locality produces a comparatively low measure of relative biodiversity when compared to other microvertebrate sites in the Mussentuchit Member using both raw and subsampling methods. Much more microvertebrate research is necessary to understand the roles of changing ecology and taphonomy that may be linked to transgression of the Western Interior Seaway or microhabitat variation.

## Introduction

The Cedar Mountain Formation, which spans both the uppermost Lower and lowermost Upper Cretaceous, records an important interval in the history of life coinciding with global climatic changes (Behrensmeyer et al., 1992) such as rising sea level (Haq, Hardenbol & Vail, 1987; Hallam, 1992; Fassell & Bralower, 1999; Frakes, 1999), global warming (Barclay, McElwain & Sageman, 2010), and the emergence of flowering plants (Crepet & Friis, 1987; Upchurch & Wolfe, 1987; Saward, 1992). Regionally, subduction of the Farallon Plate beneath North America’s west coast initiated the Sevier Orogeny—a north–south mountain range spanning from Canada to Mexico—that produced an eastward migrating forebulge (Currie, 1998; White, Furlong & Arthur, 2002; Eberth et al., 2006) followed by full inception of a foreland basin. These regional tectonic changes, coupled with global trends, resulted in the establishment of the North American Cretaceous Western Interior Seaway (WIS; Dickinson & Snyder, 1978; DeCelles, 1986; Neilson, 1986; Kauffman & Caldwell, 1993), a large, epieric body of water that flooded much of central North America by the early Late Cretaceous. Initially, rise of the Sevier Orogeny produced a period of increasing aridity that is recorded throughout most of the Cedar Mountain Formation (e.g., Yellow Cat through Ruby Ranch members) (Stokes, 1944; Young, 1960; Suarez et al., 2014; Hatzell, 2015). However, continued transgression of the WIS produced a dramatic climate shift in the capping Mussentuchit Member of the Cedar Mountain Formation, which records a rapidly changing paleoclimatic shift toward warmer, more humid conditions (Cifelli et al., 1997; Suarez et al., 2012, 2014).

Coupled with a shifting climate regime, animals inhabiting western North America during this interval also experienced a prolonged period of intercontinental faunal dispersal. Establishment of a land bridge across Beringia permitted faunal exchange between Asia and North America (Russell, 1993). This exchange has been linked to replacement of the endemic North American fauna by clades originating in Asia (Ostrom, 1970; Russell, 1993, 1995; Cifelli et al., 1997; Kirkland et al., 1997, 1999; Sereno, 1999; Nydam, 2002; Ji et al., 2003; Kobayashi & Azuma, 2003; Carpenter, 2006; Ji, Ji & Zhang, 2009; McDonald et al., 2010a; Zanno & Makovicky, 2011). Thus, paleofaunal assemblages of the Mussentuchit Member are key for recording the effects of climate change and faunal exchange across the Early to Late Cretaceous boundary in North America.

Previous microvertebrate studies of the Mussentuchit Member have documented the remains of more than 90 species (Gardner, 1994, 1996, 1999b; Cifelli et al., 1997, 1999; Fiorillo, 1999; Gardner & Cifelli, 1999; Kirkland et al., 1999, 2016; Nydam, 1999, 2000a, 2000b, 2002; Goldberg, 2000; Nydam & Cifelli, 2002; Cifelli, 2004; Garrison et al., 2007; McDonald et al., 2017), illuminating one of the most diverse Mesozoic assemblages in North America sampled to date. These taxa were discovered through extensive screen-washing and microvertebrate sampling during the latter part of the 20th century by the Oklahoma Museum of Natural History (Cifelli, 1996; Cifelli, Madsen & Larson, 1996; Cifelli et al., 1997, 1999) with a smaller effort aimed at studying the microvertebrates from a single dinosaur locality by the College of Eastern Utah (Garrison et al., 2007). Microvertebrate data collection continues through ongoing research by the North Carolina Museum of Natural Sciences and the Field Museum of Natural History.

Here, we report the taxonomic composition of a newly discovered site preserving an abundance of microvertebrate remains in the Mussentuchit Member—the Cliffs of Insanity (COI) microvertebrate locality. This site occurs in a restricted section of outcrop to the west of currently sampled localities, capturing microhabitat variation that adds critical information to the standing biodiversity during this transition period and new information on the timing and pattern of Laurasian exchange. We compare the abundance, biodiversity, and composition of the COI microvertebrate site to those from previous collection efforts. The methods used herein combine those from various fields of ecology in order to apply a more comprehensive quantitative approach to microvertebrate paleoecology.

### Geologic setting

**Figure 1:**
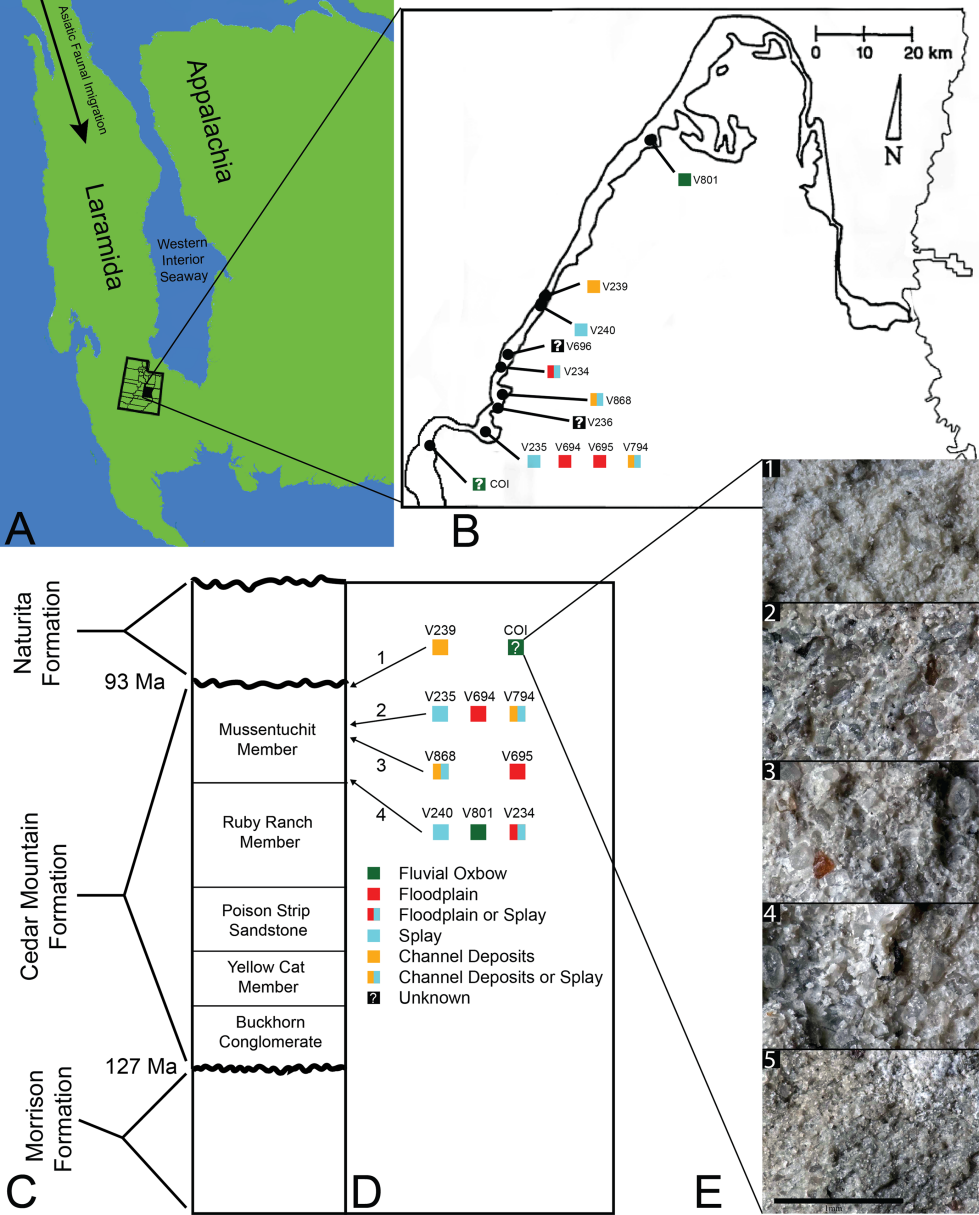
Index map with stratigraphic and geographic context. (A) Map of North America during the Cenomanian showing migration path of Asian Fauna (modified from Early Cenomanian 1; *Neogastroplites haasi*; 98.3 Ma; Blakey, 2014). (B) Map of Emery County and part of Sevier County, showing the relative locations of the COI and 11 OMNH localities. Colored squares represent inferred depositional environments. Map modified and derived from Cifelli et al. (1999) and Goldberg (2000). (C) Simplified stratigraphic cross section of the Cedar Mountain Formation with overlying and underlying formations. (D) Relative stratigraphic positions of the COI and nine OMNH localities. (1) Near the top, just below the Naturita Formation, (2) above the ash layer, (3) immediately below the ash layer, (4) near the bottom, above the contact with the Ruby Ranch Member. Colored squares represent inferred depositional environments. (E) Stratigraphic section of COI ∼1 m high, represented by five geologic samples. (1) Above fossil-bearing layer, (2) top of fossil-bearing layer, (3) middle of fossil-bearing layer, (4) lower transition layer, (5) below fossil-bearing layer. Scale bar equals one millimeter.

The Cedar Mountain Formation is stratigraphically bounded by the Upper Jurassic Brushy Basin Member of the Morrison Formation below and by the Upper Cretaceous Naturita Formation above (Kirkland et al., 1997, 1999; Carpenter, 2014). The formation is divided into six members spanning the Barremian/Aptian through the Cenomanian. Sedimentologically, the Mussentuchit Member consists predominantly of highly smectitic mudstone (formed from altered volcanic ash), containing localized, low grade coal beds (Kirkland et al., 1997, 1999) and has been interpreted as either a wet, lacustrine environment (Stokes, 1944, 1952; Craig, 1981; Garrison et al., 2007; Ludvigson et al., 2015) or a fluvial environment (Kirkland et al., 1997; Goldberg, 2000); more specifically, as a distal delta system (Sorensen, 2011; Holmes, 2017) at the western margin of the WIS in what is now central Utah (Fig. 1A). Radioistopic dates reported by Cifelli et al. (1997), Garrison et al. (2007), and Tucker, Makovicky & Zanno (in press) support a Cenomanian—early Turonian age (98.2 ± 0.6 to 93 Ma) for the Mussentuchit Member.

The microvertebrate fossils described here were recovered from a fine-grained sandstone sandwiched between siltstone-dominated units, approximately five meters below the contact with the overlying Naturita Formation. Five geologic samples above, within, and below the fossil layer were collected along a section ∼1 m high (Fig. 1E). These samples indicate the COI microfossil assemblage displays a coarsening upward sequence from siltstone below the fossil-bearing layer to a bentonitic, fine-grained sandstone within it and then a transition back to siltstone above the fossil-bearing layer, possibly representing an oxbow lake with an adjacent river migrating towards the lake. Precise locality information for this site is recorded at the NC Museum of Natural Sciences.

Microvertebrate sites published in Cifelli et al. (1999, fig. 2) indicate that the stratigraphically highest OMNH sites occur at about 13 and 17 meters below the contact with the Naturita Formation (named the Dakota Formation in the aforementioned reference). Site OMNH V824 may occur closer to the Naturita Fm.; however, Cifelli et al. (1999) state that the contact is covered in the field area of their localities so its exact distance below the contact is uncertain. The COI locality is therefore located considerably higher in section than OMNH sites previously reported, raising the likelihood of greater influence from the transgressing WIS.

## Materials and Methods

### Screenwashing and recovery

Approximately 183 kg of in situ sediment was collected from a fossiliferous horizon ∼15 m in width and 30 cm thick. The sediment, composed principally of a gray, highly bentonitic mudstone, was screenwashed in loads of 1,000 g using nylon paint sieves and traditional nested sieves (Avrahami, Heckert & Martin, 2015). A series of aquarium air bubblers was used in conjunction with the sieves to expedite breakdown of the matrix. Approximately 95% of the matrix broke down in water (99–100% breakdown of bentonite, with any remaining weight usually constituting fossils or sand). Concentrate from the paint sieves was size-sorted in nested sieves (4, 2, 1, and 0.5 mm aperture). The smallest mesh size used during screenwashing was number 35 mesh, with aperture diameters of 0.50 mm. This dimension is similar to the mesh size used to screen the 12 microvertebrate fossil assemblages considered in our comparative biodiversity analyses (0.59 mm, Cifelli, 1996), thus we suggest the assemblages are broadly comparable with regard to size of specimens sampled. Additionally, some surface collecting did occur at the COI; however, the vast majority of fossil material was obtained via quarrying-disaggregation methods. Specimens were imaged on a Keyence VHX-1000E microscope. A total of ∼6,339 fossils were recovered in the year 2014 from state lands in Utah under permit Utah 2014-447 and are reposited at the North Carolina Museum of Natural Sciences.

It should be noted that the fossil density analyses may not reflect a true assessment of each locality due to institutional variation in specimen recovery. One hundred percent of the recovered fossils from the COI were counted and catalogued by the NCSM, whereas this same approach was not applied for the specimen counts reported in Goldberg (2000). In other words, no full, actual accounting of individual fossils exists for the OMNH sites (R. Cifelli, 2018, personal communication). However, in our biodiversity analyses of the COI locality we did not include materials unidentifiable to family level, such as vertebrate bone fragments, and specimens unidentifiable to family level were also likely to have been discarded during collection of the OMNH sites, rendering our biodiversity sampling more comparable. We also used subsampling to account for differences in collection and curation protocols between institutions (see Paleoecology below).

### Taxonomic referral and morphometrics

Taxonomic referrals are based on comparisons with previously collected specimens from the Mussentuchit Member of the Cedar Mountain Formation housed at the OMNH and a variety of comprehensive microfaunal studies. We take a conservative approach, using apomorphy-based identifications to assign specimens to the most inclusive taxonomic level possible. Fragmentary or poorly preserved material lacking autapomorphies was referred using morphological similarities and assigned to higher taxonomic levels. Dental terminology follows a number of authors depending on faunal group. These can be found in supporting descriptions and references therein.

Paleoecological studies of theropods are often difficult to conduct due to the rarity of their skeletal remains, which is in some part attributable to the small size and fragility of many theropod skeletons. By comparison, theropod teeth are more mechanically and chemically resistant, and often occur in abundance at microfossil localities. Therefore, paleobiological and faunal studies that utilize isolated teeth can provide more faunal information than those that rely strictly on skeletal elements (Rensberger & Krentz, 1988; Currie, Rigby & Sloan, 1990; Farlow et al., 1991; Fiorillo, 1999; Sankey, 2001, 2008a, 2008b; Heckert, 2004; Vullo, Neraudeau & Lenglet, 2007; Schoch et al., 2018). Furthermore, due to the wide morphological diversity of theropod teeth, they can be used to identify new taxa (Currie, Rigby & Sloan, 1990; Baszio, 1997a, 1997b; Sankey, 2001; Sankey et al., 2002; Larson, 2008; Gates & Scheetz, 2015). Some theropod clades that were present in the Mussentichit Member such as oviraptorosaurs (Makovicky, Zanno & Gates, 2015), or likely present such as ornithomimosaurs, were toothless and thus would not be represented in microvertebrate samples.

**Figure 2:**
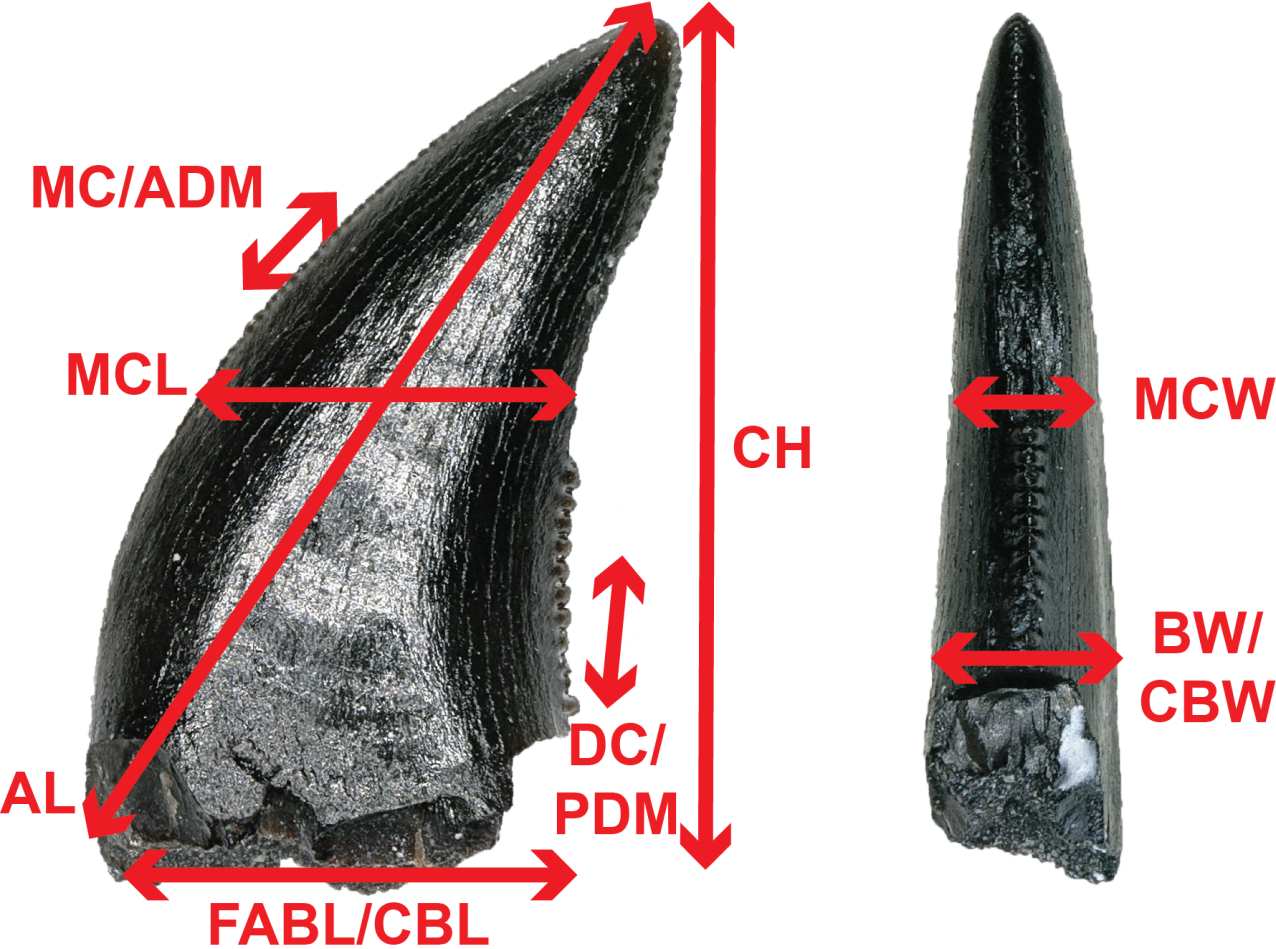
Anatomical abbreviations. All linear measurements and ratios used in the multivariate analyses. ADM, anterior denticles per millimeter; AL, apical length; BW, basal width; CBL, crown basal length; CBR, crown base ratio; CBW, crown basal width; CH, tooth crown height; CHR, crown height ratio; DC, distocentral denticle density; FABL, fore aft basal length; MC, mesiocentral denticle density; MCL, mid-crown length; MCR, mid-crown ratio; MCW, mid-crown width; PDM, posterior denticles per millimeter.

To better constrain the diversity of theropod species represented in our sample, we followed the approach of several recent studies (Smith, Vann & Dodson, 2005; Larson & Currie, 2013; Hendrickx, Mateus & Araújo, 2014; Williamson & Brusatte, 2014; Gates & Scheetz, 2015) by performing principal component (PCA) and discriminant (LDA) analyses in the program PAST version 3.19 (Hammer, Harper & Ryan, 2001) on two, independently derived, taxonomically comprehensive databases of theropod tooth measurements including the eight theropod teeth from the COI. Measurements follow Smith, Vann & Dodson (2005) and Larson & Currie (2013) (Fig. 2) and were taken in the program ImageJ (Rasband, ImageJ & National Institutes of Health, 2011). Both PCA and LDA identify the hypothetical variables (components) that account for the majority of variation in a multivariable dataset by transforming the new variables into linear combinations of the original variables (Davis, 1986; Harper, 1999). An LDA is a supervised analysis, which discriminates according to class, whereas a PCA is an unsupervised analysis that ignores class labels (Martínez & Kak, 2001).

We performed an LDA using a taxonomically comprehensive dataset (Hendrickx, Mateus & Araújo, 2014) that includes 11 principal measurements (CBL, CBW, CH, AL, MCL, MCW, MC, DC, CBR, CHR, and MCR) (Data S1). The Hendrickx, Mateus & Araújo (2014) dataset comprises 995 teeth assigned to one of 16 qualitative morphotype categories, each representing a clade, family, paraphyletic group, or a theropod species of uncertain affinity. All values were log-transformed to better reflect a normally distributed multivariate dataset. Three theropod teeth from the COI were recovered with missing apices. To account for this uncertainty, we estimated a maximum CH for each tooth (Data S1). Estimated maximum CHs were plotted in independent analyses in order to maintain statistical integrity, and resultant data points were then superimposed onto the output of prior analyses. Thus, the true point resides somewhere along the line, most likely close to the estimated CH point.

We performed a second series of PCA and LDA using a novel combination of numerical data derived from Larson & Currie (2013) and Williamson & Brusatte (2014) (sampled from Late Cretaceous North America theropods in contrast to the Hendrickx, Mateus & Araújo (2014) dataset, which sampled widely across the Mesozoic theropod diversity), then removed samples missing a measured FABL, CH, or BW (Fig. S1). These samples were removed because FABL, CH, and BW often represent the strongest lodgings in the multivariate analyses and there was uncertainty regarding how this missing data would be handled. The five principal measurements included in the combined database are FABL, CH, BW, PDM, and ADM. The modified dataset consists of 1,027 teeth belonging to one of six taxa (including two tooth morphotypes) identified to species by previous authors. These categories include Dromaeosauridae, *Paronychodon*, *Richardoestesia*, Saurornitholestinae, Troodontidae, and Tyrannosauroidea. Teeth were further separated into a total of 14 categories based on lithostratigraphic unit and measurement data that was log-transformed to better reflect a normally distributed multivariate dataset. This independent analysis was performed in order to check and contrast the results of the LDA using the Hendrickx, Mateus & Araújo (2014) database. However, the Hendrickx, Mateus & Araújo (2014) database is superior in terms of theropod diversity, tooth completeness, confidence of taxonomic identifications, and number of measured principal components. Therefore, we consider the LDA results using the Hendrickx, Mateus & Araújo (2014) database more heavily in our taxonomic identifications than those of our other analyses.

### Histology

We produced and examined paleohistological sections of isolated turtle shell to corroborate taxonomic referrals made from gross morphology. Three specimens of adocids and three specimens of helochelydrids, formerly referred to as Solemyidae (Joyce, Sterli & Chapman, 2014), were sampled. Specimens were embedded in synthetic resin and ten thin sections were produced following standard procedures for the preparation of petrographic thin sections (Scheyer et al., 2007). Sections were cut using a Buehler IsoMet 1000 Precision Saw and studied using a Nikon Eclipse Ci POL microscope equipped with an Iphone 7 camera.

### Paleoecology

In order to contrast taphonomic and ecologic signals between microvertebrate localities within the Mussentuchit Member, we added the faunal information from the COI locality to the Goldberg (2000) dataset of 12 OMNH sites, then reduced taxonomic data to family-level and higher, using actual counts as opposed to simple presence-absence (Data S2). Next, we used this database to calculate three quantities: fossil densities, the proportion of the total number of fossils that each locality contributed to the dataset, and the Shannon–Wiener biodiversity index (Shannon & Weaver, 1949; Spellerberg & Fedor, 2003). Fossil density for each site was calculated as the *total number of fossils from a locality divided by the total sediment weight sampled from that site*. This is not, strictly speaking, a density because volume was not in the equation, but total weight of sediment is the most often provided measurement for the amount of sediment sampled from a microvertebrate locality. The proportion of fossils in the dataset was calculated as the *total number of fossils from each locality divided by the total number of fossils included in the entire database*. The proportion of fossils provides a measure of influence for each site when assessing the pie chart of total diversity from microvertebrate localities, rarefaction curves, and Shannon–Wiener biodiversity index calculated for the entire dataset.

Due to variations in collection and cataloging methods between the NCMNS and the OMNH, in addition to the raw Shannon–Wiener indices calculated for each locality, we also created several datasets in which we subsampled the COI locality to a sample size equal to each of the OMNH fossil localities. Subsampling and subsequent calculation of biodiversity indices was carried out in R version 3.5.0 (R Core Team, 2015) using the vegan package v. 2.5-2 (Oksanen et al., 2013). Within the *sample* function of R, probability weights for the selection of each taxon was accomplished by simply taking the proportion of a given clade within the known COI database. This means that if a clade was not present in the COI site then it received a zero probability of being selected in the subsampled dataset. The resulting COI subsampled dataset was then used to calculate the Shannon–Wiener index. Afterward, we subtracted the OMNH locality index value from the subsampled COI index in order to assess the difference in biodiversity calculation between the two sites. Difference in Shannon–Wiener values is the quantity of interest in our case, not the calculated value of each subsampled COI index. Instead we focus on the comparison between the COI and a respective OMNH site (whose Shannon–Wiener index does not change), which requires the difference in values. This process of subsampling, recalculating the Shannon–Wiener index, and determining the difference was repeated 100 times for each OMNH locality. Additionally, since the sample sizes between the COI site and the OMNH sites were equal in the subsampled datasets, we calculated the Morisita–Horn similarity index in order to assess how similar the subsampled COI fossils were to the original OMNH preserved faunas. Again, this calculation was performed 100 times per OMNH locality, except using the R package fossil v. 0.3.7 (Vavrek, 2011). Complete results for these analyses and the R code used to derive them are available in the Supplementary Materials.

Rarefaction curves were produced to assess the likelihood of recovering additional higher-level taxa from each locality. As mentioned, the database used for this calculation is based on family-level taxonomy and above. As such, the rarefaction is calculating the likelihood of finding new representatives at those taxonomic levels, not at the species level. Both the Shannon–Wiener diversity indices and the rarefaction curves were produced using PAST (version 3.19; Hammer, Harper & Ryan, 2001).

Finally, to assess the impact of geology and stratigraphic position on the preserved fauna, we used multivariate techniques to draw comparisons with the other vertebrate microfossil sites in the Mussentuchit Member included in Goldberg (2000). We produced a correspondence analysis (CA) in PAST version 3.19 (Hammer, Harper & Ryan, 2001), using the same faunal data from the paleoecology analyses (Data S2). CA is a multivariate ordinal technique used to explore relationships among a matrix of categorical or continuous variables (Hammer, Harper & Ryan, 2001), and has been used by other studies to understand diversity changes across gradients (Gates et al., 2010), because of the method’s robust nature to missing data (Hirst & Jackson, 2007). A total of 11 of the 12 OMNH microsites were associated with one of four types of depositional environment, categorized by Goldberg (2000) as either fluvial oxbow, floodplain, crevasse splay, or channel deposits. In our analysis, each site was categorized according to Goldberg’s assessment whereas COI was categorized as an ox-bow lake, in accordance with our independently derived geologic interpretation. These category labels were then overlain on the plotted data to qualitatively assess the role of depositional environment on preserved faunal remains.

## Results

First, we present our taxonomic assessment of the assemblage in a traditional “Systematic Paleontology” format, followed by subsequent analyses of biodiversity, paleoecology, and paleoenvironmental influences, based on comparisons of the COI assemblage with other microvertebrate assemblages from the Mussentuchit Member.

## Systematic Paleontology

OSTEICHTHYES Huxley, 1880
ACTINOPTERYGII Klein, 1885
NEOPTERYGII Regan, 1923

**Figure 3:**
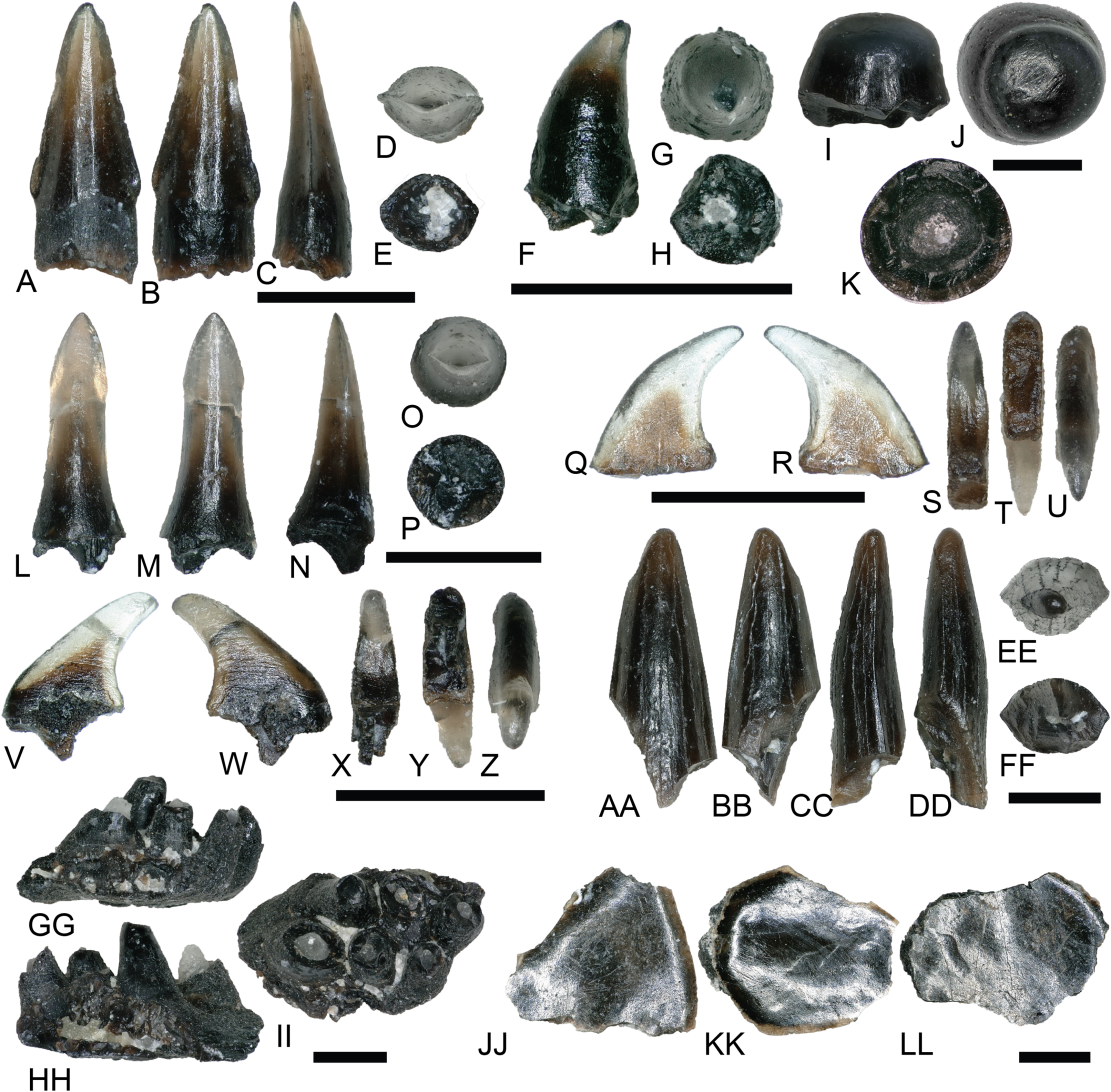
Osteichthyan material. (A–E) (NCSM 33292) Amiid tooth in (A) lingual, (B) labial, (C) mesial?/distal?, (D) occlusal, and (E) basal views. (F and G) (NCSM 33279) Lepisosteiform marginal tooth in (F) mesial?/distal?, (G) occlusal, and (H) basal views. (I–K) (NCSM 33280) Lepisosteiform vomerine tooth in (I) mesial?/distal?, (J) occlusal, and (K) basal views. (L–P) (NCSM 33282) Lepisosteiform tooth in (L) labial, (M) lingual, (N) mesial?/distal?, (O) occlusal, and (P) basal views. (Q–U) (NCSM 33304) Pycnodontiform tooth in (Q–R) labial?/lingual?, (S) distal, (T) basal, and (U) occlusal views. (V–Z) (NCSM 33303) Pycnodontiform tooth in (V–W) labial?/lingual?, (X) distal, (Y) basal, and (Z) occlusal views. (AA–FF) (NCSM 33307) Enchodontid tooth fragment in (AA) labial, (BB) lingual, (CC–DD) mesial?/distal?, (EE) occlusal, and (FF) basal views. (GG–II) (NCSM 33297) Fish spines in (GG–HH) mesiodistal/labiolingual and (II) dorsal views. (JJ–LL) (NCSM 33361) fish scales. Scale bars equal one millimeter.

Approximately 112 teeth (Figs. 3A–3FF), 53 complete or partial scales (Figs. 3JJ–3LL), and additional skeletal and vertebral fragments were recovered representing Osteichthyes. Amiiform and lepisosteiform teeth constitute the majority of material, whereas only three teeth represent pycnodontids. Fish material unidentifiable to lower taxonomic levels are shown in Figs. 3GG–3LL.

AMIIFORMES Hay, 1929 (sensu Grande & Bemis, 1998)
AMIINAE Bonaparte, 1838

Amiid teeth from the COI possess a suite of characteristics typical of Amiidae as outlined by Estes & Sanchíz (1982), including a styliform morphology with a rounded crown situated atop a bony pedestal (Figs. 3A–3E). Well-developed carinae extend from the crown base to the apex and the teeth become increasingly transparent toward the apex similar to those described by Bryant (1988).

LEPISOSTEIFORMES Hay, 1929
LEPISOSTEIFORMES indent

Over 56 partial scales attributable to gars were discovered at the COI site, as is common for many nonmarine Cretaceous microvertebrate fossil concentrations. For taxonomic assignment we followed Grande (2010), Brinkman et al. (2013), and Brinkman, Newbrey & Neuman (2014) in considering isolated scales as generically indeterminate.

LEPISOSTEIDAE Cuvier, 1825

There are three morphotypes of lepisosteiform teeth from the COI. Two of these were originally described by Bilelo (1969; but see also Estes & Sanchíz, 1982; Fiorillo, 1999) as dentary and palatal teeth of lepisosteids from the Lower Cretaceous Paluxy Formation of north-central Texas, and a third, lanceolate tooth type was described by Brinkman et al. (2013) from the Upper Cretaceous (Campanian) Kaiparowits Formation and the Upper Cretaceous John Henry Member of the Straight Cliffs Formation. McDonald et al. (2017) illustrated the first gar tooth from the Mussentuchit Member, which has striking similarities to specimens in our locality such as those in Figs. 3L–3P. Dentary (marginal?) teeth are short, broad, and conical with a smooth, translucent crown, and are occasionally falcate (Figs. 3F–3H). Teeth of the palatine are hemispherical with a smooth, convex crown (Figs. 3I–3K). The third tooth type accounts for the majority of recovered lepisosteiform material and is similar to the first type in that they are conical and translucent. However, the crowns possess elongated, narrow peduncles that constrict below the apex (Figs. 3L–3P). The apex is distinguished by short, well-developed, mesio-distal carinae.

PYCNODONTIFORMES Berg, 1940

Pycnodontiform teeth from the COI are strongly falcate, with a smooth, translucent crown (Figs. 3Q–3Z). They are strongly labiolingually compressed and lack any striations or a carinae. They are identical to pycnodont teeth recovered at the Cifelli #2 *Eolambia caroljonesa* Quarry in Mussentuchit Member Wash of Utah (Garrison et al., 2007) as well as other localities across the Cretaceous of North America (Brinkman et al., 2013).

SALMONIFORMES Greenwood, Rosen, Weitzman, and Myers, 1966
cf. ENCHODONTIDAE Lydekker, 1889

NCSM 33308 may represent an *Enchodus* tooth fragment (Figs. 3AA–3FF), sharing a suite of features with those described in Fiorillo (1999) and Winkler, Murry & Jacobs (1990). The tooth fragment is slender and labiolingually compressed with a lenticular cross section in basal view. It is slightly recurved, and the apex is blunt and rounded. The enamel is smooth and thin, lacking striations, and there are weakly defined carinae on the mesial and distal margins. The fragmentary nature of NCSM 33308 makes an accurate referral to *Enchodus* tenuous, however, the presence of three *Enchodus* teeth that have been previously reported from the Mussentuchit Member (OMNH 026333, 026983, and 032537) may add support for this referral.

LISSAMPHIBIA Haeckel, 1866
ALLOCAUDATA Fox and Naylor, 1982
ALBANERPETONTIDAE Fox and Naylor, 1982
*ALBANERPETON* Estes and Hoffstetter, 1976
ALBANERPETON

**Figure 4:**
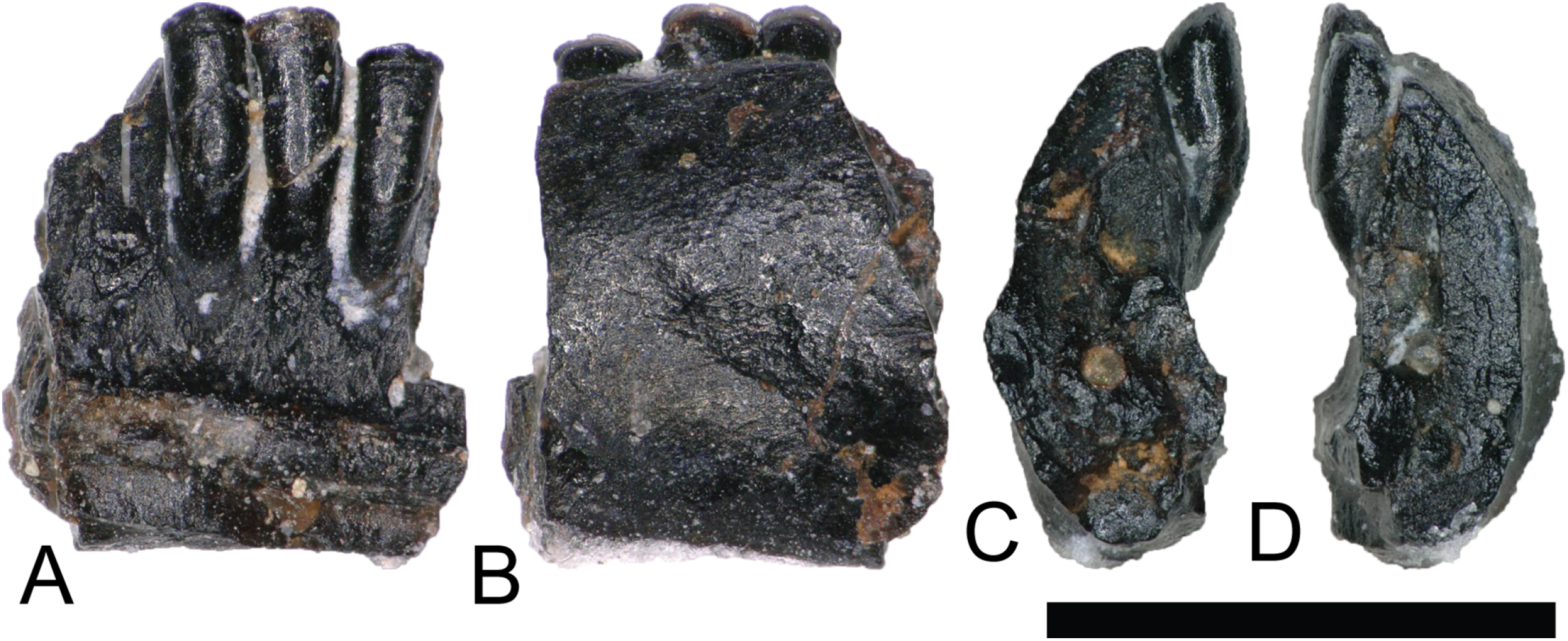
*Albanerpeton* dentary. (NCSM 33278) Lisamphibian dentary fragment likely belonging to a species of *Albanerpeton* in (A) medial, (B) lateral, and (C and D) cross section views. Scale bar equals one millimeter.

NCSM 33278 (Fig. 4) is a lissamphibian dentary bearing three complete teeth and one tooth fragment. Much of the jaw is missing and the lack of diagnostic elements makes assigning it to a lower taxonomic level difficult. However, Gardner (1999a) describes several dental characteristics of *Albanerpeton arthridion* that NCSM 33278 shares. These include teeth with strong labiolingual compression that are non-pedicellate, chisel shaped, and with strong pleurodont implantation. Additionally, teeth are straight and arranged closely together along the parallel orientations of their lengths. Oreska, Carrano & Dzikiewicz (2013) describe *Albanerpeton* material from the Lower Cretaceous Cloverly Formation resembling NCSM 33278.

REPTILIA Linnaeus, 1758
TESTUDINATA Klein, 1760
HELOCHELYDRIDAE (SOLEMYDIDAE) Lapparent de Broin and Murelaga, 1996

**Figure 5:**
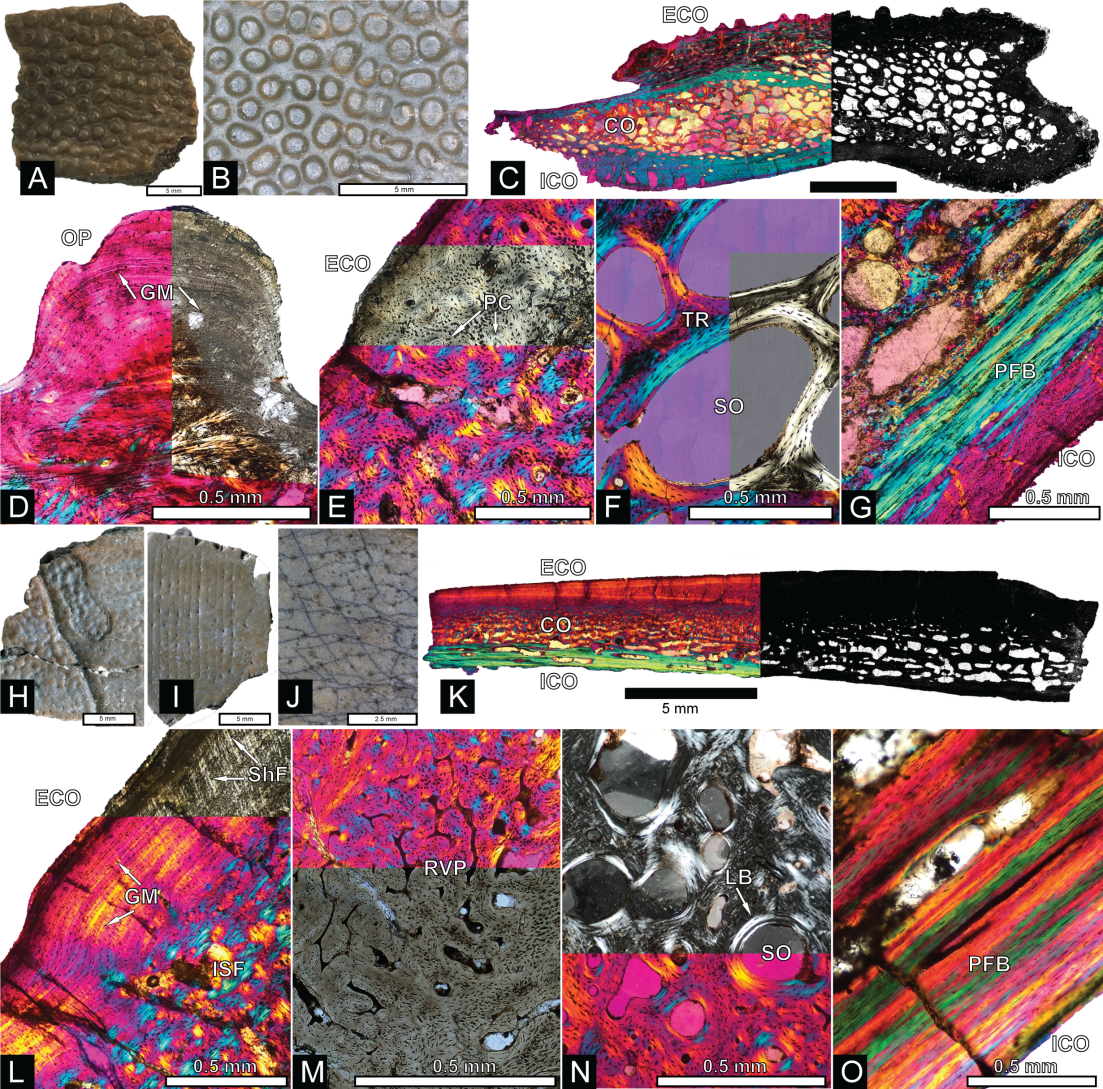
Helochelydra and adocidae shell histology. (A–G) Helochelydra. (H–O), Adocidae. (A–B) (NCSM 33383) shell fragment showing external ornamentation pattern. (C) (NCSM 33380) Sectioned Helochelydra specimen in polarized light and thresholded indicating internal bone density. (D) Close-up of ornamentation in polarized light. (E) (NCSM 33388) Close-up of the ECO in polarized light. (F) (NCSM 33380) close-up of the CB showing trabeculae and SO, in polarized light. (G) (NCSM 33388) close-up of the internal cortex showing PFB, in polarized light. (H–J), shell fragments showing external sculpting pattern (NCSM 33387, NCSM 33382). (K) (NCSM 33381) Sectioned Adocidae specimen in polarized light and thresholded indicating internal bone density. (L) (NCSM 33387) Close-up of ECO in polarized light with ISF in the lower zone and GM and ShF in the upper zone. (M) close-up of the RVP in normal transmitted light and polarized light. (N) (NCSM 33381) close-up of the CB showing SO with lamellar bone around the internal walls, in polarized light. (O) (NCSM 33387) close-up of the internal cortex showing PFB, in polarized light. Abbreviations: CB, cortical bone; ECO, external cortex; GM, growth mark; ISF, interwoven structural fiber bundles; OP, ornamentation pattern; PC, primary vascular canal; PFB, parallel-fibred bone; RVP, reticular vascularization pattern; SO, secondary osteon; ShF, Sharpey’s fibers. Scale bars equal one millimeter unless otherwise noted.

The majority of turtle materials from the COI are referable to a new species of helochelydrid, previously referred to *Naomichelys speciosa* (Herzog, Zanno & Makovicky, 2015). Helochelydrid shell fragments, characterized by a dense arrangement of raised, cylindrical tubercles, are common throughout the Mussentuchit Member (Herzog, Zanno & Makovicky, 2015). Tubercles are typically 0.5–1 mm tall with planar apices and constricted bases (Figs. 5A and 5B). Microstructurally, COI helochelydrid shell samples are similar to *Solemys vermiculata* and *Solemys* sp. previously reported from Spain, and *Naomichelys* sp. previously reported from Canada and USA (Scheyer, Pérez-García & Murelaga, 2015). All samples possess a dipole structure with cortices of relatively equal thickness (Fig. 5C). The external cortex is characterized by an outer zone of ornamental tubercles composed of parallel-fibered bone (Fig. 5D). The lower zone of the external cortex is composed of coarse, interwoven structural bone fibers (Fig. 5E). The cancellous bone is comprised of long slender trabeculae with large intertrabecular regions (Fig. 5F). Secondary lamellar bone is often present along the walls of the trabeculae. The internal cortex is made of parallel fibered bone and occasionally grades into lamellar zonal bone (Fig. 5G).

ADOCIDAE Cope, 1870

The COI contains shell fragments likely representing a new species of adocid (Figs. 5H–5O). Danilov, Sukhanov & Syromyatnikova (2011) describes adocid shell sculpturing as having a diagnostic arrangement of small and regular grooves and pits or dots. COI shell fragments ascribed to Adocidae are similar to those described by Danilov, Sukhanov & Syromyatnikova (2011) and Danilov et al. (2013) and are characterized by a smooth surface with small pits and rhomboidal sulci. A few shell fragments are sculptured, bearing small grooves and pits (Figs. 5H–5J). The identification of these specimens as adocid is supported by similarities of internal microstructure (Scheyer, Syromyatnikova & Danilov, 2017). All samples possess thick, densely packed external and internal cortical bone with well-developed interior cancellous bone (Fig. 5K). The external cortex is divided into upper and lower zones. The lower zone (bordering the internal cancellous bone) is made of interwoven structural fibers and is characterized by a reticular vascular pattern. The upper zone (bordering the external surface) is characterized by highly birefringent growth marks exhibiting a wavy pattern, and diagonally oriented Sharpey’s fibers (Fig. 5L). The cancellous bone is less spongy than found in *Helochelydra*, and is made of coarse, dense, short trabeculae with round to oval intertrabecular regions (Fig. 5N). Secondary lamellar bone is often present along the walls of the trabeculae. The internal cortex is made of parallel-fibered bone and occasionally grades into lamellar zonal bone (Fig. 5O).

SQUAMATA Oppel, 1811

**Figure 6:**
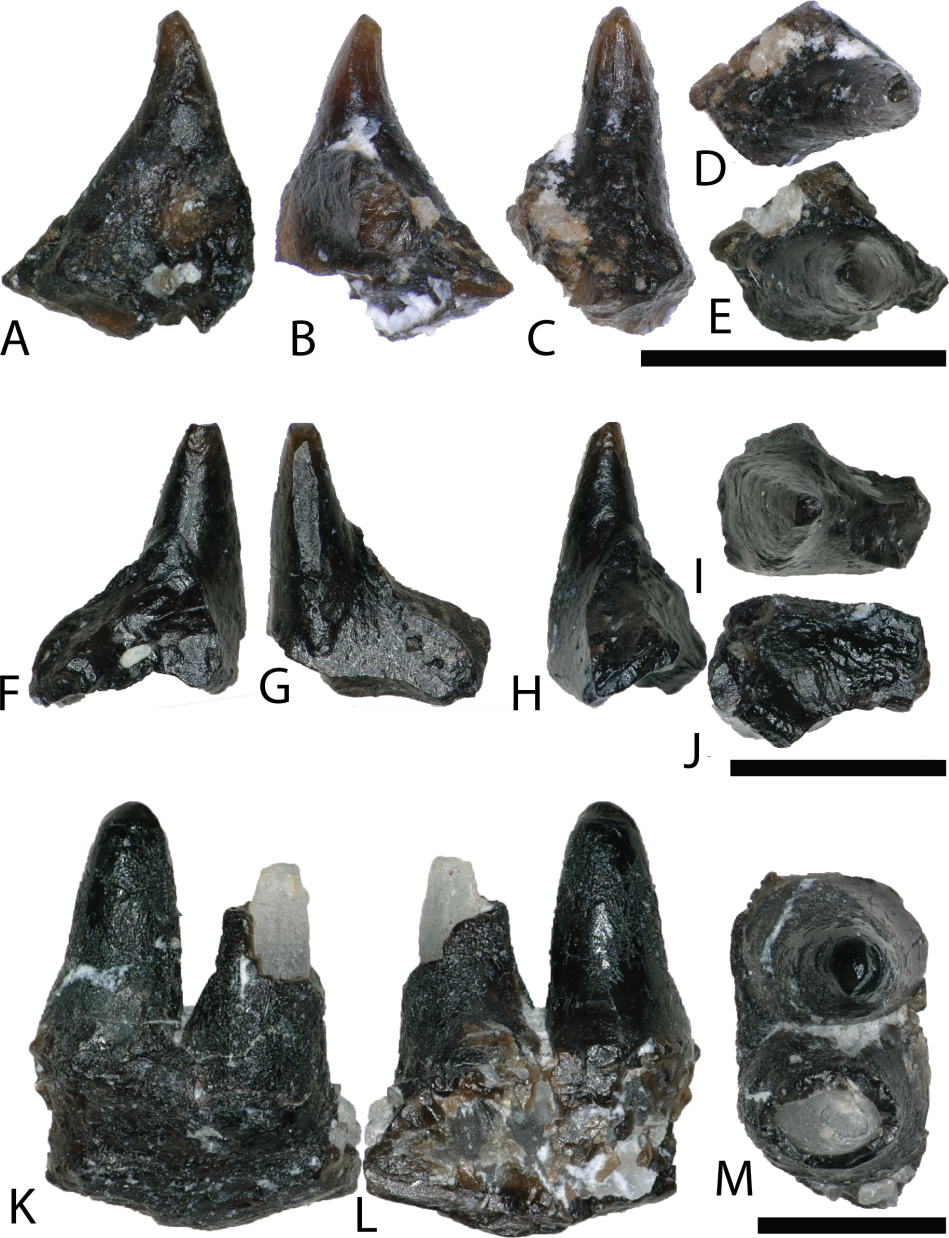
Squamata teeth. (A–E) (NCSM 33293) scincomorphan tooth in (A) mesial?/distal?, (B) mesial?/distal?, (C) lingual, and (D and E) occlusal views. (F–J) (NCSM 33295) Possible squamate tooth in (F–H) mesiodistal?/labiolingual?, (I) occlusal view, and (J) basal view (K–M) (NCSM 33296) Possible scincomorphan jaw fragment in (K and L) medial?/lateral? view, and (M) dorsal view. Scale bars equal one millimeter.

Squamata is represented by two isolated teeth (Figs. 6A–6J) and a jaw fragment bearing two teeth (Figs. 6K–6M). The poorly preserved and fragmentary condition of these specimens makes confident referrals to lower taxonomic levels difficult, however they share a suite of characteristics with squamate dental material described from the Mussentuchit Member (Nydam, 2002) and other Cretaceous-age sedimentary rocks (Nydam, Rowe & Cifelli, 2013).

SCINCOMORPHA Camp, 1923

NCSM 33293 is a scincomorphan (paramacellodid–cordylid grade) (R. Nydam, 2018, personal communication) with subpleurodont implantation and deposits of cementum at the base (Figs. 6A–6E). The tooth is tall, narrow, conical, slightly recurved distally, with a weakly defined carina and several weakly developed striations on the lingual crown surface.

SQUAMATA indet.

NCSM 33295 is a poorly preserved, isolated tooth possibly belonging to Squamata (Figs. 6F–6J). The tooth is tall, narrow, conical, lacks curvature, and is subpleurodont with heavy deposits of cementum at the base.

SQUAMATA indet.

NCSM 33296 is a possible scincomorphan jaw fragment with two closely placed teeth (R. Nydam, 2018, personal communication) (Figs. 6K–6M). The complete tooth is tall, conical, lacks curvature, has heavy deposits of cementum at the base. The enamel is poorly preserved, improving slightly toward the tooth crown. The tooth crown is rounded and blunt, with a weakly defined carina.

ARCHOSAURIA Cope, 1869
CROCODYLIFORMES Hay, 1930, sensu Benton and Clark, 1988
MESOEUCROCODYLIA Whetstone and Whybrow, 1983

**Figure 7:**
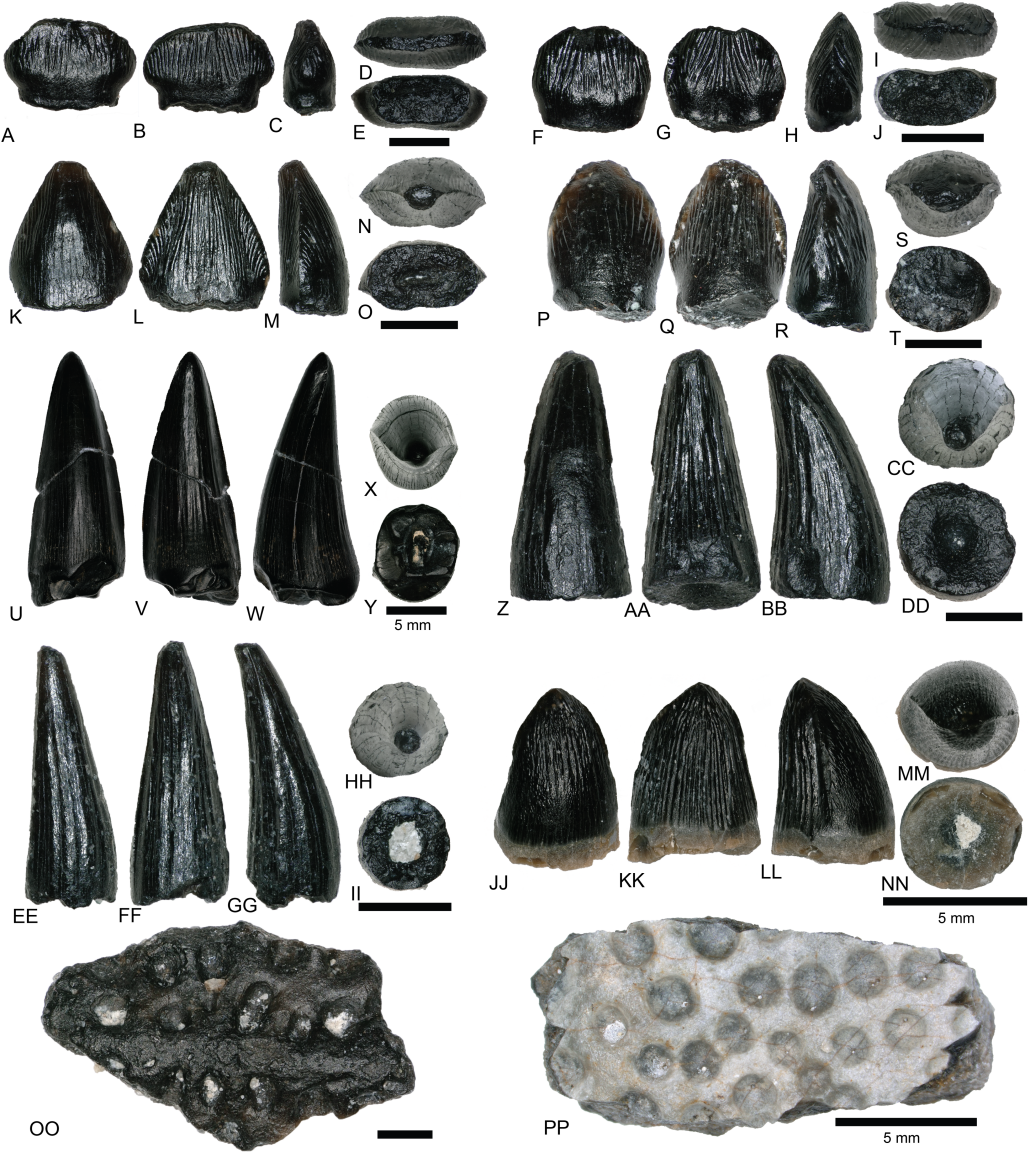
Mesoeucrocodylia teeth and osteoderms. (A–E) (NCSM 33284) Bernissartid-like tooth in (A) labial, (B) lingual, (C) mesial?/distal?, (D) occlusal, and (E) basal views. (F–J) (NCSM 33286) Bernissartid-like tooth in (F) labial, (G) lingual, (H) mesial?/distal?, (I) occlusal, and (J) basal views. (K–O) (NCSM 33305) atoposaurid-like tooth in (K) labial, (L) lingual, (M) mesial?/distal?, (N) occlusal, and (O) basal views. (P–T) (NCSM 33315) atoposaurid-like tooth in (P) labial, (Q) lingual, (R) mesial?/distal?, (S) occlusal, and (T) basal views. (U–Y) (NCSM 33269) *Dakotasuchus* teeth in (U) labial, (V) lingual, (W) mesial?/distal?, (X) occlusal, and (Y) basal views. (Z–DD) (NCSM 33290) Pholidosaurid-like tooth in (Z) labial, (AA) lingual, (BB) mesial?/distal?, (CC) occlusal, and (DD) basal views. (EE–II) (NCSM 33289) Pholidosaurid-like tooth in (EE) mesial?/distal?, (FF) lingual, (GG) mesial?/distal?, (HH) occlusal, and (II) basal views. (JJ–NN) (NCSM 33270) Pholidosaurid-like teleosaurid tooth in (JJ) labial, (KK) lingual, (LL) mesial?/distal?, (MM) occlusal, and (NN) basal views. (OO–PP), (NCSM 33362, NCSM 33384) mesoeucrocodylia osteoderms. Scale bars equal one millimeter unless otherwise noted.

By far the most abundant identifiable material from the COI is crocodyliform (Fig. 7), constituting approximately 3,657 teeth (∼94% of all teeth) as well as abundant osteoderm fragments. We note similarities of these teeth to four Late Cretaceous groups (bernissartids, atoposaurids, pholidosaurids (Pomes, 1988), and cf. *Dakotosuchus*). However, these assignments should be considered tentative as there is a great variability within and between tooth morphologies, with many teeth possessing a suite of characteristics along a gradational spectrum (Buscalioni et al., 2008). Following the example of McDonald et al. (2017), we simply consider the morphotypes under Mesoeucrocodylia with no further speculation to their taxonomic framework. Similarly, this approach was applied for all biodiversity analyses.

MESOEUCROCODYLIA indet.

Teeth we identify as similar in morphology to bernissartids are apico-basally short, mesio-distally elongate, labiolingually compressed, and slightly reniform in occlusal view (Figs. 7A–7J). The crowns often possess an elliptical apical wear facet. The mesial and distal margins have poorly defined carinae and the labial and lingual faces are ornamented with widely spaced, well-defined, apicobasally oriented striations that converge toward the apex. Teeth matching this description have been recovered from the Lower Cretaceous Cloverly Formation of Wyoming and Montana and the Lower Cretaceous Rabekke, Jydegård, and Annero formations of Scandinavia (Schwarz-Wings, Rees & Lindgren, 2009; Oreska, Carrano & Dzikiewicz, 2013).

Teeth resembling atoposaurids from the COI are proportionately taller, labiolingually compressed, and reniform in occlusal view (Figs. 7K–7T). They are constricted toward the crown base and have well-developed, sometimes crenulated, mesial, and distal carinae. They are typically triangular in mesio-distal view, with a strongly convex labial face and a weakly convex lingual face. Tooth crowns range from strongly rounded to moderately acuminate and have varying degrees of recurvature. Striations run apicobasally, are sometimes anastomosing, and are typically more pronounced on the lingual face. Recent descriptions of similar teeth have come from the Lower Cretaceous Cloverly Formation of Wyoming and Montana and other sites in the Mussentuchit Member of the Cedar Mountain Formation (Garrison et al., 2007; Oreska, Carrano & Dzikiewicz, 2013; McDonald et al., 2017).

Teeth morphologically similar to those of pholidosaurids are considerably taller, strongly conical, and have nearly circular bases (Figs. 7Z–7NN). Smaller teeth are tall and slender, with slight lingual recurvature and sharp apices (Figs. 7Z–7II). Larger teeth tend to be more stout with rounded apices (Figs. 7JJ–7NN). Mesial and distal carinae extend from the base to the apex and are weakly defined. Striations are strongly defined and run apicobasally, sometimes anastomosing towards the apex. Larger, stouter teeth have numerous, thin, well defined striations that run apicobasally (Figs. 7JJ–7NN). These larger teeth have been previously reported from the Cedar Mountain Formation as a fifth group of teleosaurid teeth similar to *Machimosaurus* (Cifelli et al., 1997), which display a nearly complete sinusoidal curve outlined by the primary carinae in apical view (R. Nydam, 2018, personal communication).

NCSM 33362 and NCSM 33384 are mesoeucrocodylian osteoderms (Figs. 7OO and 7PP). Due to breakage there is no evidence of an anterior bar or posterior suture to aid identification and so we assign these osteoderms to Mesoeucrocodylia indet. NCSM 33384 possesses a random array of approximately equal-sized round pits, generally similar to the morphology on pholidosaurids and goniopholids. One feature on NCSM 33362 is a low median keel that barely rises above the level of the dermal body (Fig. 7OO).

cf. *DAKOTASUCHUS* sp. Mehl (1941)

Teeth we identify as similar to *Dakotasuchus* are conical, sometimes slightly lingually recurved, and typically sub-circular in occlusal view (Figs. 7U–7Y) (Frederickson et al., 2017). They are somewhat labiolingually compressed, with the labial side slightly more convex than the lingual. Tooth bases are circular to somewhat elliptical and the crown apices are strongly pointed. Both the mesial and distal carinae extend from the base to the apex and are less pronounced than those of similar to atoposaurid-like teeth. Enamel ornamentation is characterized by multiple, weakly defined, apicobasally oriented, parallel striations. Teeth of *Dakotasuchus* are morphologically similar to goniopholid and pholidosaurid teeth (Garrison et al., 2007; Puértolas-Pascual, Rabal-Garcés & Canudo, 2015; Frederickson et al., 2017).

DINOSAURIA Owen, 1842
SAURISCHIA Seeley, 1887
THEROPODA Marsh, 1881

**Figure 8:**
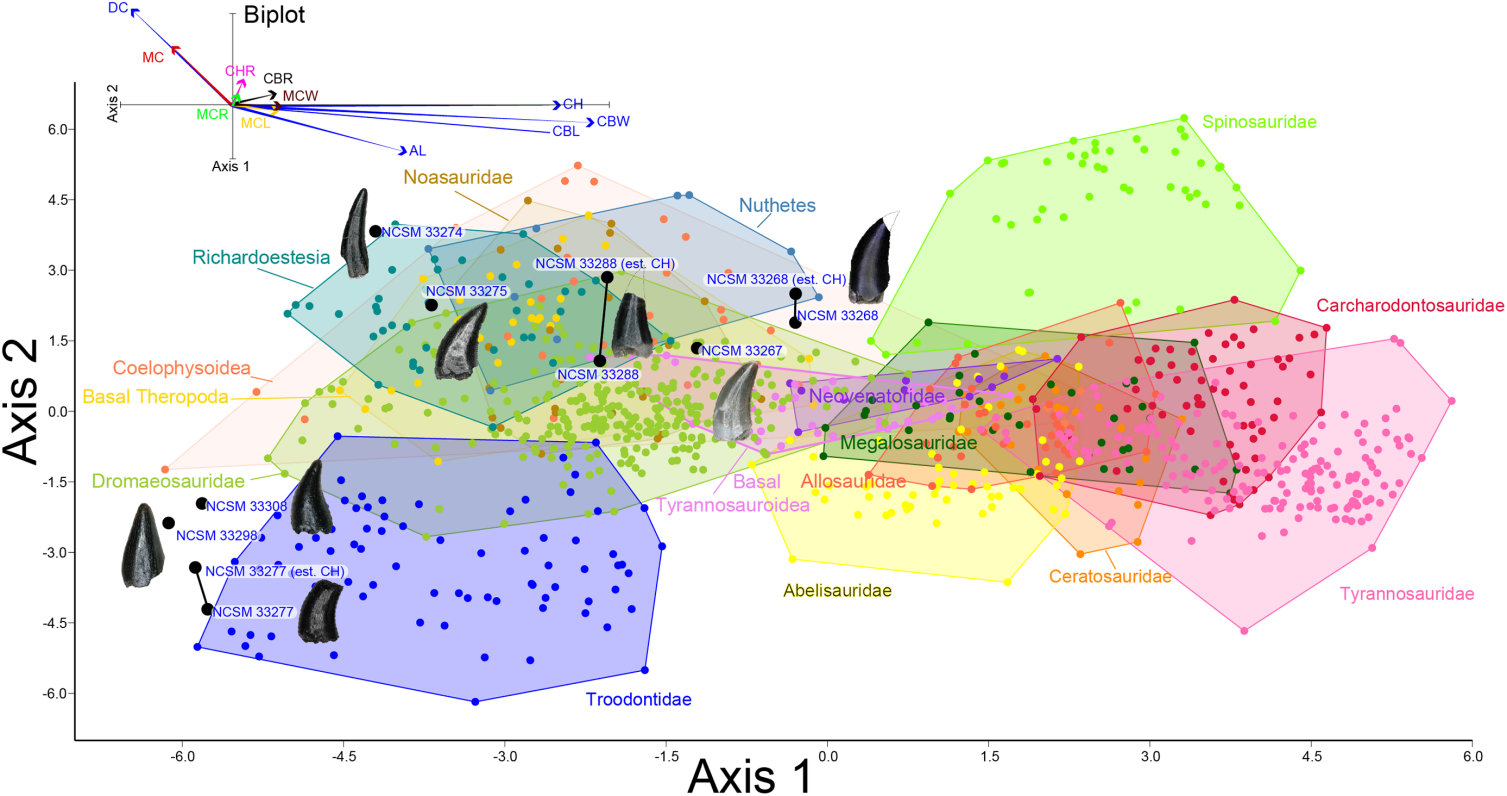
Linear discriminant analysis of theropod teeth. Multivariate analysis of eight theropod teeth from the COI and 994 theropod teeth from Hendrickx, Mateus & Araújo (2014). All linear measurements were log-transformed. The biplot and eigenvectors represent the direction and magnitude of each linear measurement and ratio.

Theropods from the COI are represented by eight complete to partially complete teeth, 20 tooth fragments, and 11 bone fragments. Tooth and bone fragments are too poorly preserved to allow for confident lower level taxonomic identifications. Therefore, only the eight well-preserved teeth are described here. These eight teeth are identified to varying degrees of taxonomic confidence based on qualitative features and the results of the morphological analyses (Fig. 8). Qualitative tooth descriptions are based on Hendrickx, Mateus & Araújo (2015).

COELUROSAURIA Von Huene, 1914
COELUROSAURIA indet.

**Figure 9:**
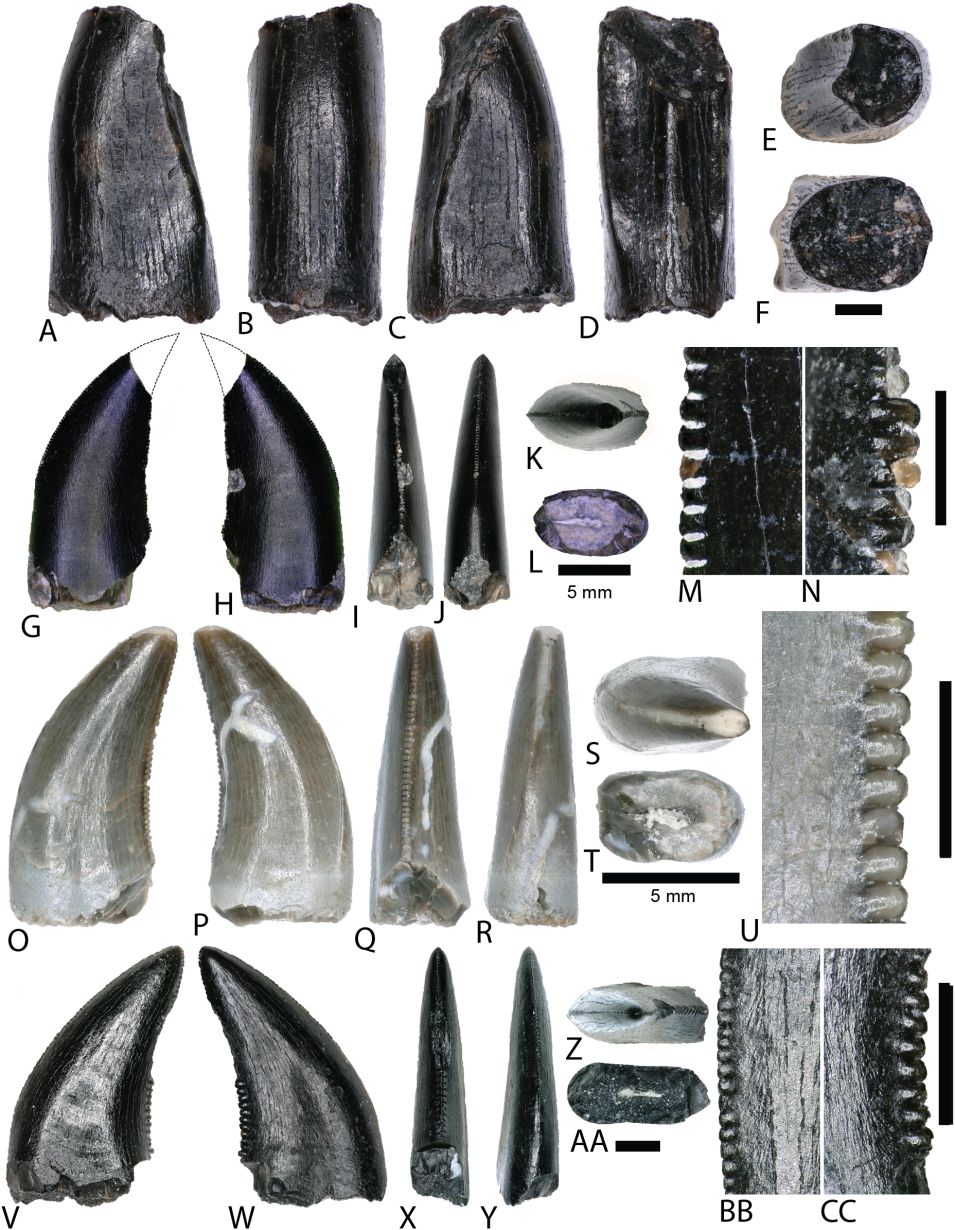
Tyrannosaur and dromaeosaur teeth. (A–F) (NCSM 33276) tyrannosaurid premaxillary tooth in (A) mesial?/distal?, (B) labial, (C) mesial?/distal?, (D) lingual, (E) occlusal, and (F) basal views. (G and H) (NCSM 33268) tyrannosaurid? tooth in (G) lingual, (H) labial, (I) distal, (J) mesial, (K) occlusal, and (L) basal views, with close-ups of mesial (M) and distal (N) denticles. (O–U) (NCSM 33267) dromaeosaur tooth in (O) lingual, (P) labial, (Q) distal, (R) mesial, (S) occlusal, and (T) basal views, with close-up of mesial (U) denticles. (V–CC) (NCSM 33275) dromaeosaur tooth in (V) lingual, (W) labial, (X) distal, (Y) mesial, (Z) occlusal, and (AA) basal views, with close-ups of mesial (BB) and distal (CC) denticles. Scale bars equal one millimeter unless otherwise noted.

NCSM 33268 (Figs. 9G–9N) is the largest theropod tooth recovered from the COI, with a preserved crown height of 17.5 mm, an estimated crown height of ∼20 mm, a FABL of 8.39 mm, and a basal width of 4.5 mm. The exact crown height is unknown because the apical portion of the tooth is missing. The tooth is robust, lacks recurvature in mesial view, and possesses a wide elliptical basal cross section. The enamel texture is veined and apicobasally oriented. Both the anterior and posterior denticles are subrectangular in shape and centrally positioned. The mesial carinae becomes unserrated towards the base as is typical with tyrannosaurids (Buckley et al., 2010). When considered as part of the Hendrickx, Mateus & Araújo (2014) database (Fig. 8), NCSM 33268 plots within the Coelophysoidea and *Nuthetes* morphospaces, the former being an improbable identification for a Late Cretaceous theropod and the latter, an enigmatic theropod of indeterminate affinity, but likely referable to dromaeosaurids or tyrannosauroids (Sweetman, 2004; Rauhut, Milner & Moore-Fay, 2010). However, in the PCA and LDA performed with the combined (Larson & Currie, 2013; Williamson & Brusatte, 2014) database NCSM 33268 consistently plots within or adjacent to Tyrannosauroidea and Dromaeosauridae (Fig. S1).

TYRANNOSAUROIDEA Osborn, 1906

NCSM 33276 (Figs. 9A–9F) is a premaxillary tooth and the only specimen that can be confidently referred to as a tyrannosauroid based on a suite of diagnostic characteristics (Currie, Rigby & Sloan, 1990; Baszio, 1997b; Carr & Williamson, 2004; Zanno & Makovicky, 2011; Williamson & Brusatte, 2014). These include a D-shaped cross section, a lingually oriented carina that arches around the lingual side of the tooth, a greater arc length along the labial face than the lingual face, a lack of denticles and serrations, and a prominent ridge on the lingual surface. The tooth has a crown height of 6 mm and part of the lingual surface, near the apex is worn away.

MANIRAPTORA Gauthier, 1986
DROMAEOSAURIDAE Matthew and Brown, 1922

Two teeth are assigned to Dromaeosauridae from the COI. Diagnostic characteristics of dromaeosaurid teeth are outlined in Currie, Rigby & Sloan (1990) and Sankey et al. (2002). Maxillary and dentary teeth are laterally compressed and recurved in lateral view, lack basal constriction, and possess a flattened, oval cross section. Distal denticles are thin, sometimes point apically, and larger than mesial serrations, and the distal carina is often positioned toward the lingual margin. In both datasets, the dromaeosaurid convex hulls have wide regions that overlap with Tyrannosauridae and *Richardoestesia*. Thus, several teeth may in fact belong to other groups and should be considered tentatively assigned.

NCSM 33267 (Figs. 9O–9U) has a crown height of 10.15 mm, a FABL of 5.03 mm, and a basal width of 3.32 mm. The tooth lacks recurvature in mesial view and has an incrassate figure-eight-shaped cross section (sensu Hendrickx, Mateus & Araújo, 2015). Wear facets extend along the apical portion of the distal carina and across the smooth enamel of the labial surface. The mesial carina lacks serrations and twists lingually near the base. The distal denticles are subrectangular and the distal carina is positioned towards the lingual margin. When considered as part of the Hendrickx, Mateus & Araújo (2014) database, NCSM 33267 plots exclusively within Dromaeosauridae (Fig. 8).

NCSM 33275 (Figs. 9V–9CC) has a crown height of 5.83 mm, a FABL of 3.23 mm, and a basal width of 1.36 mm. The tooth is strongly compressed laterally, lacks recurvature in mesial view, and has a weakly figure-eight-shaped cross section. The enamel texture is smooth and a depression is present on the lingual face. The mesial carina is centrally positioned and the distal carina is positioned centrally at the apex, turning toward the lingual margin near the base. Both mesial serrations and distal denticles are subrectangular. When plotted with the Hendrickx, Mateus & Araújo (2014) database NCSM 33275 plots within *Richardoestesia* and close to Dromaeosauridae (Fig. 8). However, NCSM 33275 is assigned to Dromaeosauridae because it possesses strong recurvature in lateral view, a characteristic of dromaeosaurid teeth that is not accounted for in the dataset.

*RICHARDOESTESIA* Currie, Rigby & Sloan, 1990
RICHARDOESTESIA sp.

**Figure 10:**
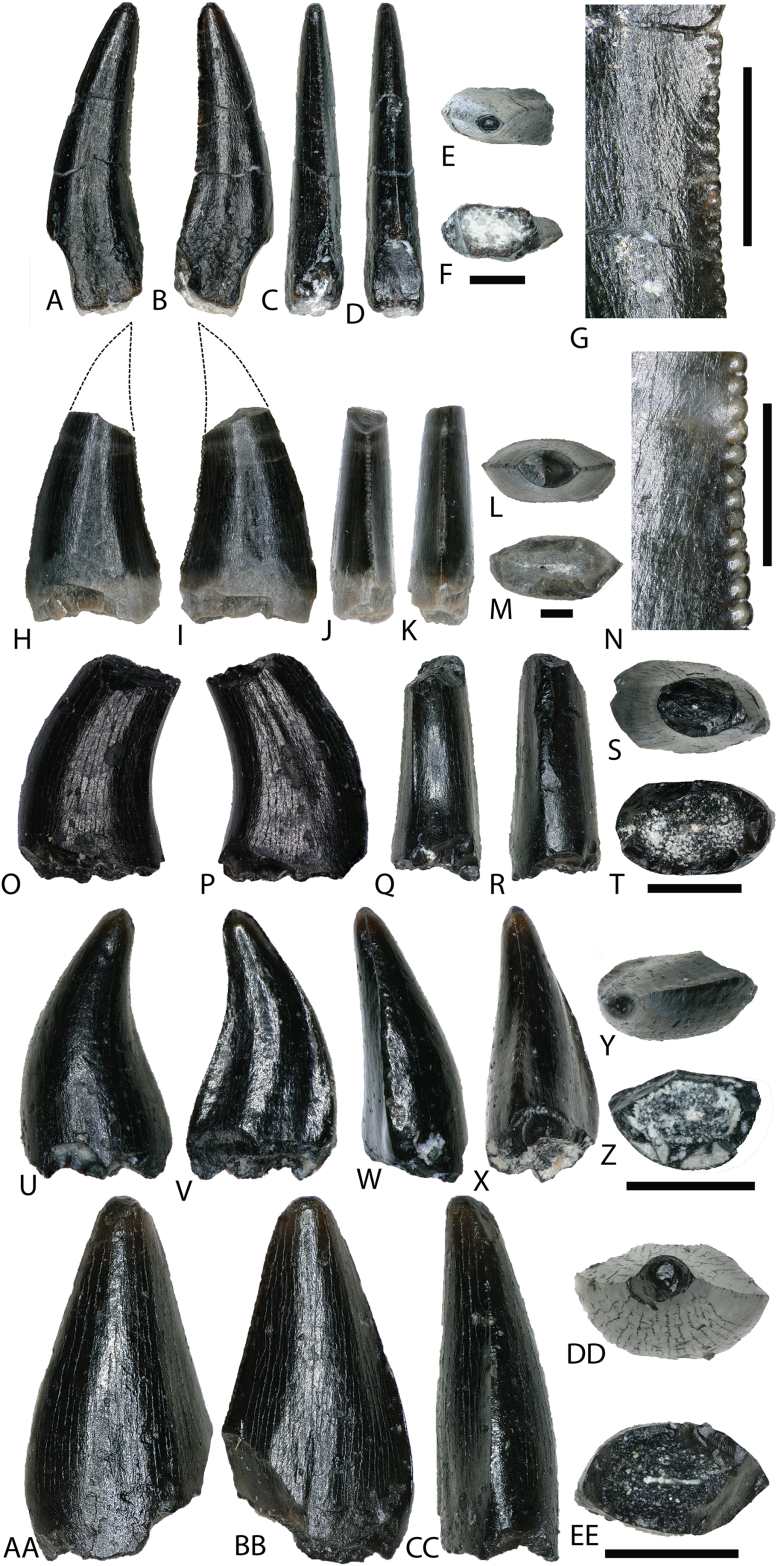
*Richardoestesia* and *Paronychodon* teeth. (A–G) (NCSM 33274) *Richardoestesia* tooth in (A) lingual, (B) labial, (C) distal, (D) mesial, (E) occlusal, and (F) basal views, with close-up of mesial (G) denticles. (H–N) (NCSM 33288) *Richardoestesia* tooth in (H) lingual, (I) labial, (J) distal, (K) mesial, (L) occlusal, and (M) basal views, with close-up of mesial (N) denticles. (O–T) (NCSM 33277) *Paronychodon* tooth in (O) lingual, (P) labial, (Q) distal, (R) mesial, (S) occlusal, and (T) basal views. (U–Z) (NCSM 33298) *Paronychodon?* tooth in (U) labial, (V) lingual, (W) mesial, (X) distal, (Y) occlusal, and (Z) basal views. (AA–EE) (NCSM 33308) *Paronychodon?* tooth in (AA) labial, (BB) lingual, (CC) mesial, (DD) occlusal, and (EE) basal views. Scale bars equal one millimeter.

NCSM 33274 (Figs. 10A–10G) is complete and includes part of the root. NCSM 33274 is ascribed to *Richardoestesia* based on a suite of characteristics outlined in Currie, Rigby & Sloan (1990; see also Sankey et al., 2002) and from the results of the LDA (Fig. 8). NCSM 33274 has a crown height of 4.21 mm, a FABL of 1.65 mm, and a basal width of 0.98 mm. The tooth is tall and slender, lacks curvature in mesial view, is weakly recurved in lateral view, and has an oval to figure-eight-shaped cross section in basal view. The mesial carina lacks serrations and is centrally positioned, whereas the distal carina is positioned centrally at the apex, turning toward the lingual margin near the base. Distal denticles are small and sub rectangular. There is a depression near the base on both the lingual and labial surfaces and the enamel has a weakly braided texture (sensu Hendrickx, Mateus & Araújo, 2015). When considered as part of the Hendrickx, Mateus & Araújo (2014) database, NCSM 33274 plots slightly outside of the *Richardoestesia* convex hull.

NCSM 33288 (Figs. 10H–10N) has a crown height of 6.58 mm, an estimated crown height of ∼9.00 mm, a FABL of 4.05 mm, and a basal width of 1.9 mm. The exact crown height is unknown because the apical ∼third of the tooth crown is missing. The tooth is laterally compressed, weakly recurved in lateral view, lacks recurvature in mesial view, and has an elliptical cross section. The enamel texture is smooth and a subtle longitudinal depression is present on the lingual face. The mesial carina is centrally positioned, lacks serrations, and is worn away except near the base. The distal carina is positioned centrally at the apex, turning slightly towards the lingual margin near the base. Denticles along the distal carina are sub-rectangular to weakly apically oriented. When considered as part of the Hendrickx, Mateus & Araújo (2014) database, NCSM 33288 plots along a line representative of reconstructed crown height. Nearly all points along the line fall within the overlapping morphospace of *Richardoestesia, Nuthetes,* and Dromaeosauridae (Fig. 8).

*PARONYCHODON* Cope, 1876
PARONYCHODON sp.

Three teeth are referred to *Paronychodon* based on a suite of characters outlined by Currie, Rigby & Sloan (1990; see also Rauhut, 2002; Sankey et al., 2002). These teeth are recurved in lateral view, with a flattened lingual face, a convex labial face, numerous longitudinal ridges, oval cross section, and lacking denticles or serrations. Previous studies have tentatively referred *Paronychodon* to Dromaeosauridae (Antunes & Sigogneau-Russell, 1991), Troodontidae (Osmólska & Barsbold, 1990), and Aves (Rauhut, 2002).

NCSM 33277 (Figs. 10O–10T) has a crown height of 2.4 mm, an estimated crown height of ∼3.3 mm, a FABL of 1.57 mm, and a basal width of 1.00 mm. The exact height is unknown because part of the tooth crown is missing. NCSM 33277 is laterally compressed, recurved in lateral view, and has an elliptical cross section in basal view. The labial face is convex and the lingual face is flattened with a weakly defined longitudinal ridge. The distal carina is positioned lingually and lacks denticles. The mesial carina is worn away except at the base, positioned lingually, and lacks serrations. The enamel texture is smooth and ornamented by numerous longitudinal striations along the lingual and labial surface. When considered as part of the Hendrickx, Mateus & Araújo (2014) dataset, NCSM 33277 plots slightly outside of the troodontid morphospace (Fig. 8).

NCSM 33298 (Figs. 10U–10Z) has a crown height of 2.11 mm, a FABL of 1.16 mm, and a basal width of 0.77 mm. It is recurved in lateral view and has an asymmetrically salinon cross section (sensu Hendrickx, Mateus & Araújo, 2015) in basal view. The labial face is strongly convex and the lingual face is flattened with a well-defined longitudinal ridge. Both the distal and mesial carinae are positioned lingually and lack denticles or serrations and the distal carina is deflected inward. The enamel texture is smooth and there is a slight constriction at the base. When considered as part of the Hendrickx, Mateus & Araújo (2014) database, NCSM 33298 plots slightly outside of the troodontid morphospace (Fig. 8).

NCSM 33308 (Figs. 10AA–10EE) has a crown height of 2.75 mm, a FABL of 1.39 mm, and a basal width of 0.85 mm. It is laterally compressed, lacks recurvature, and has a lenticular cross-section in basal view. The labial and lingual faces are convex, and the lingual face has a weakly defined medial depression near the base. The distal and mesial carinae lack denticles or serrations and the mesial carina twists lingually at the base. The enamel texture is ornamented by numerous longitudinal striations with no real relief extending baso-apically along the lingual and labial surfaces. When considered as part of the Hendrickx, Mateus & Araújo (2014) database, NCSM 33308 plots slightly outside of the troodontid morphospace (Fig. 8). NCSM 33308 may belong to Aves based on a series of characteristics specific to this tooth morphotype that are not accounted for in the analysis such as recurvature, flattened lingual surface, absence of denticles, and an oval cross section. Therefore, NCSM 33308 may be a morphologically distinct bird tooth or possibly a mesially positioned theropod tooth.

AVES Linnaeus, 1758
Gen. et sp. indet.

Bird material from this locality is difficult to properly discern, as no complete skeletal material has been documented and teeth vary widely in morphology (Sankey et al., 2002; Mayr et al., 2007; Larson & Currie, 2013; Wilson, Chin & Cumbaa, 2016). Two morphologically distinct teeth from the COI are tentatively ascribed to Avialae.

**Figure 11:**
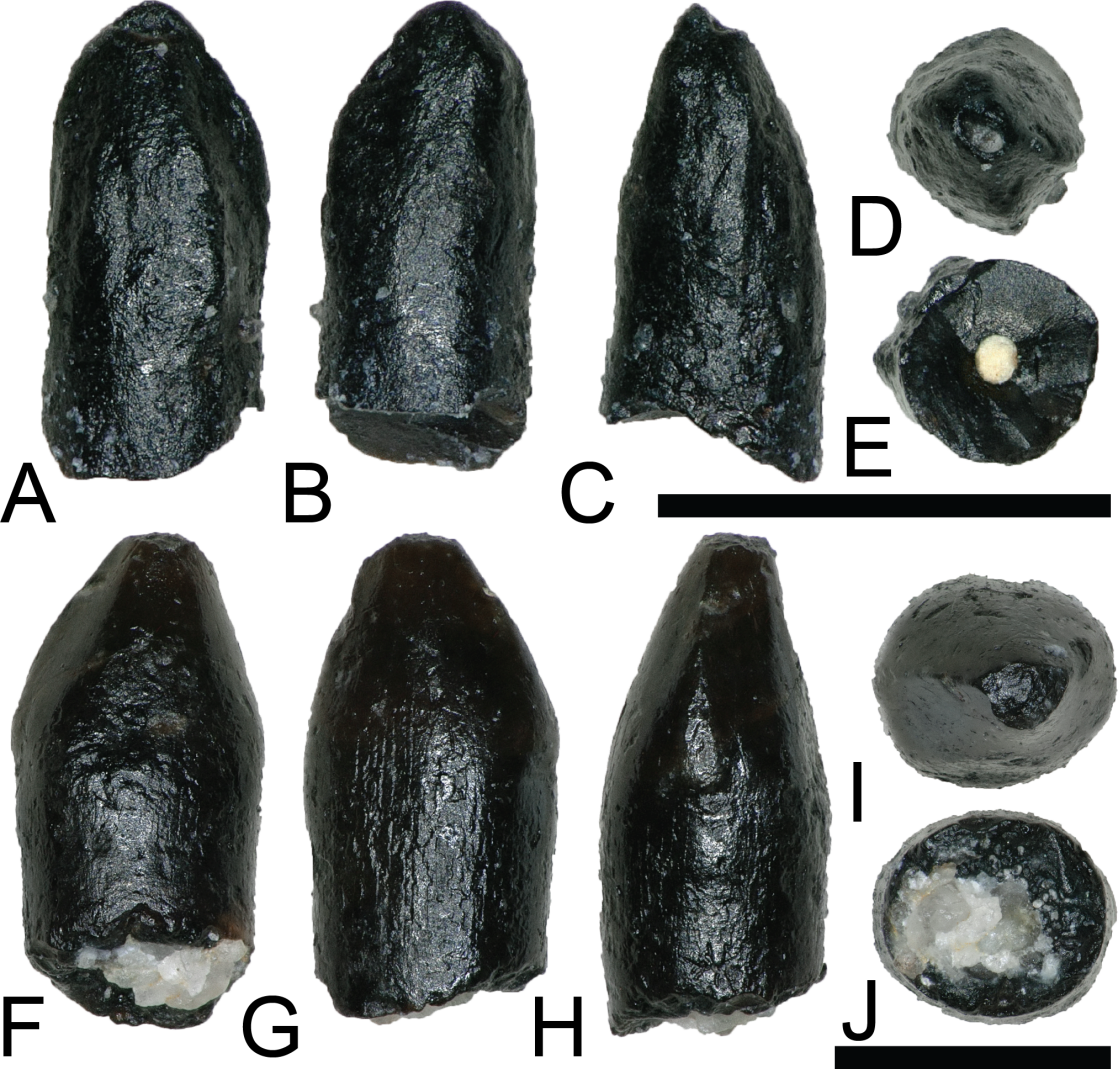
Possible avian teeth. (A–E) (NCSM 33299) Bird tooth? in (A) lingual?, (B) labial?, (C) mesial?, (D) occlusal, and (E) basal views. (F–J) (NCSM 33300) Bird tooth? in (F) labial, (G) lingual, (H) distal, (I) occlusal, and (J) basal views. Scale bars equal one millimeter.

NCSM 33299 (Figs. 11A–11E) and NCSM 33300 (Figs. 11F–11J) are similar in morphology and size to three teeth from the OMNH referred to Aves: OMNH 28735, 28736, and 28737. These teeth are very small, measuring ∼1 mm in height, are constricted at the base, and have circular cross sections. The crowns are asymmetrical, have weakly defined carinae on both anterior and posterior edges, and lack serrations or denticles. The enamel is smooth and moderately worn, and the crown apex is usually worn flat.

DINOSAURIA Owen, 1842
ORNITHISCHIA Seeley, 1887
ORNITHISCHIA indet.

**Figure 12:**
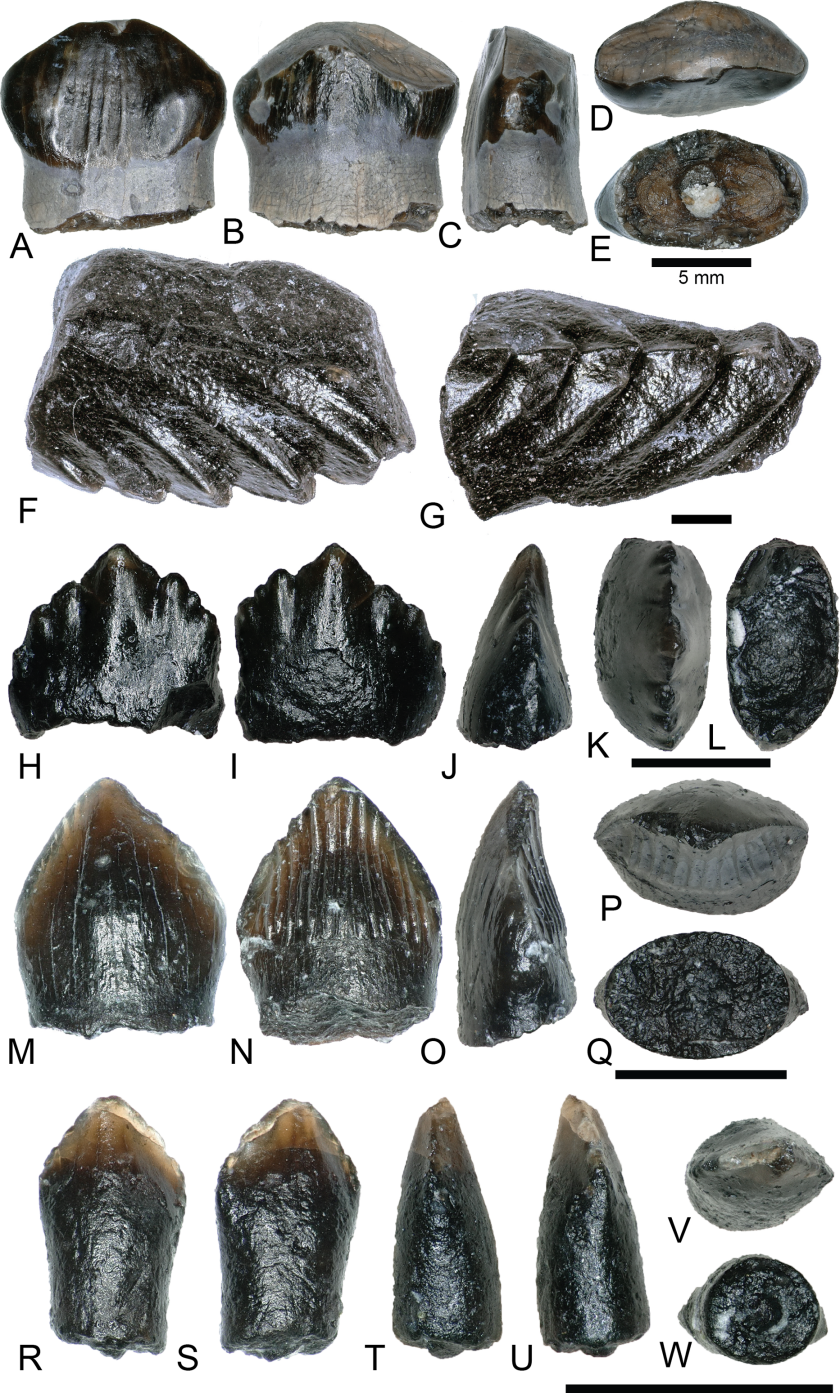
Ankylosaur and ornithischian indet. **Teeth.** (A–E) (NCSM 33322) Ankylosaur tooth in (A) lingual?, (B) labial?, (C) mesial?/distal?, (D) occlusal, and (E) basal views. (F and G) (NCSM 33318) Ankylosaur? tooth fragment in (F) labial?/lingual? and (G) mesial?/distal? views. (H–L) (NCSM 33316) Neornithischian tooth in (H) lingual, (I) labial, (J) mesial?/distal?, (K) occlusal, and (L) basal views. (M–Q) (NCSM 33312) Neornithischian? tooth in (M) labial, (N) lingual, (O) mesial?/distal?, (P) occlusal, and (Q) basal views. Scale bars equal one mm. (R–W) (NCSM 33314) Ornithischian tooth? in (R) labial?, (S) lingual?, (T and U) mesial?/distal?, (V) occlusal, and (W) basal views. Scale bars equal one millimeter unless otherwise noted.

NCSM 33322 (Figs. 12A–12E) is a heavily worn tooth with a CH of 7.31 mm, a FABL of 9.59 mm, and a BW of 5.71 mm. There is a large wear facet that extends across the apical portion of the labial surface. Even after accounting for wear, the tooth is mesio-distally elongate and apico-basally short. The tooth is constricted below the crown and several worn ridges run apicobasally along the lingual surface. Due to the poor preservation, NCSM 33322 cannot be confidently referred to a lower taxonomic level. However, NCSM 33322 compares favorably with illustrations of worn ankylosaur teeth (e.g., Coombs, 1978, fig. 20.6d). Based on the megaherbivore clades known to have inhabited the Western Interior Basin of North America during the Late Cretaceous, a referral to Ankylosauridae is the most plausible.

NCSM 33318 (Figs. 12F–12G) is a 6.8 mm long tooth fragment, preserving five large denticles. NCSM 33318 compares most favorably with ankylosaurid teeth (Coombs, 1978).

NCSM 33316 (Figs. 12H–12L) is a small, triangular tooth with a CH of 1.4 mm, a FABL of 1.49 mm, and a BW of 0.79 mm, possibly pertaining to Neornithischia, Ankylosauria, or Pachycephalosauria. It has seven cusps, a typical characteristic of premaxillary teeth of basal euornithopods (Oreska, Carrano & Dzikiewicz, 2013), and lacks an elevated rim along with the primary and secondary ridges present in ornithopod teeth such as *Zalmoxes* (Virág & Ősi, 2017).

NCSM 33312 (Figs. 12M–12Q) is possibly the premaxillary tooth of a neornithischian (Fanti & Miyashita, 2009), with a CH of 0.95 mm, a FABL of 1.18 mm, and a BW of 0.73 mm. It is a small, triangular, scoop-shaped tooth with a constriction below the crown. The labial face is smooth and strongly convex. The lingual face is concave and possesses ∼15 well defined, apicobasally oriented ridges.

NCSM 33314 (Figs. 12R–12W) may represent a small ornithischian tooth, with a CH of 0.45 mm, a FABL of 0.55 mm, and a BW of 0.34 mm. It has a triangular crown that is weakly constricted between the base and root, and appears to have seven weakly defined denticles and apicobasally oriented striations.

ORNITHOPODA Marsh, 1881
HADROSAUROIDEA Cope, 1863

**Figure 13:**
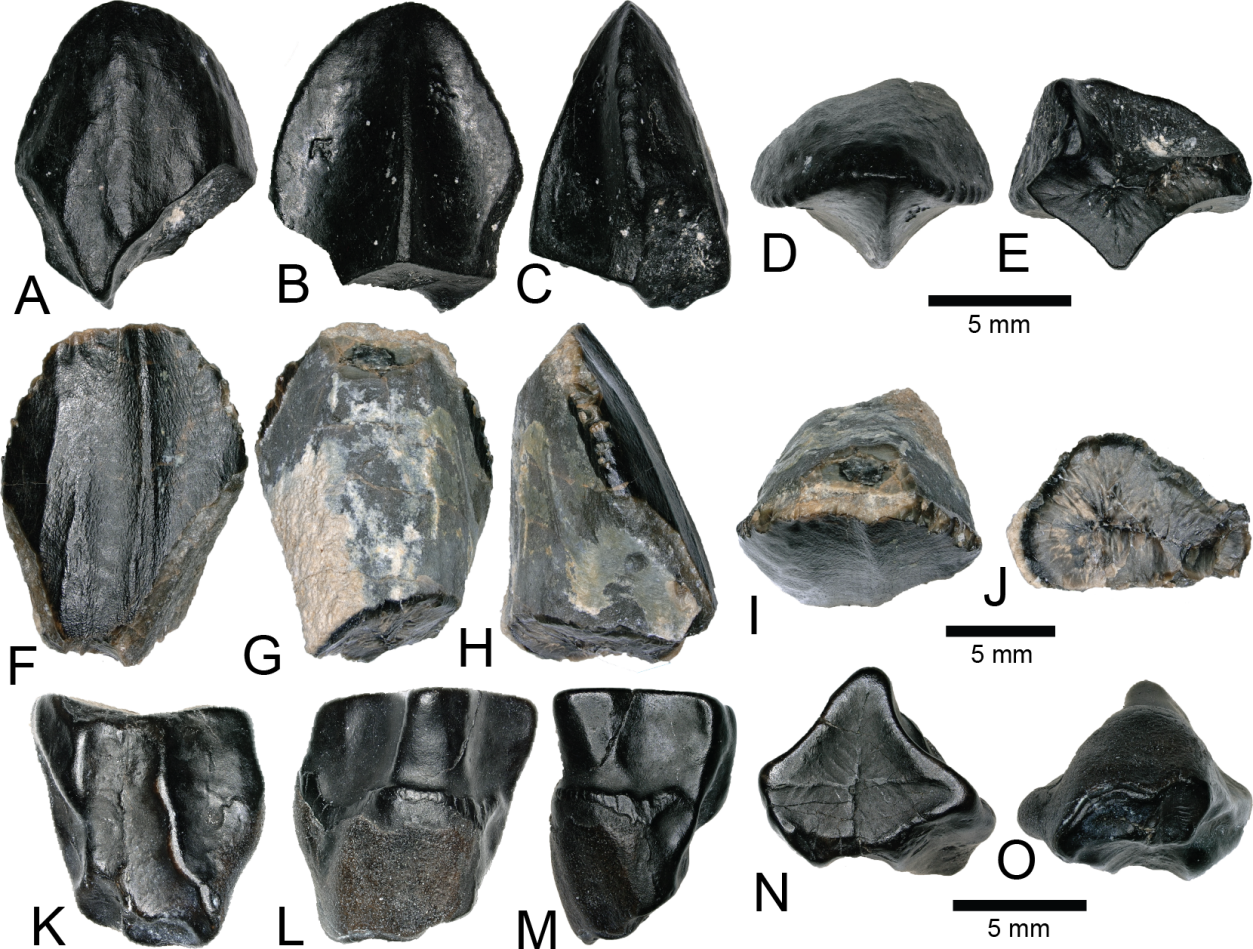
Hadrosaurid teeth. (A–E) (NCSM 33320) Possible *Eolambia* tooth in (A) labial, (B) lingual, (C) mesial?/distal?, (D) occlusal, and (E) basal views. (F–J) (NCSM 33321) Possible *Eolambia* tooth in (F) labial, (G) lingual, (H) mesial?/distal?, (I) occlusal, and (J) basal views. (K–O) (NCSM 33323) Possible *Eolambia* tooth in (K) labial?, (L) lingual?, (M) mesial?/distal?, (N) occlusal?, and (O) basal? views. Scale bars equal five millimeters.

NCSM 33320, NCSM 33321, and NCSM 33323 (Figs. 13A–13O) are hadrosauroid teeth possibly referable to *Eolambia caroljonesa*, the only hadrosauroid yet described from the Mussentuchit Member (Cifelli et al., 1997; Kirkland, 1998; McDonald et al., 2017). The teeth show considerable wear and are marked by linear and branched tubule ridges along the transverse cross section. These tubules produce multiple shearing faces along the chewing surface (Erickson et al., 2012). Each tooth possesses a prominent main keel, and NCSM 33321 (Figs. 13F–13J) shows two additional smaller ridges running nearly parallel to the primary keel. Remnants of denticles on the apical edge of NCSM 33320 and NCSM 33321 demonstrate the presence of these features, but they are too worn to make comparisons.

MAMMALIA Linnaeus, 1758
METATHERIA Thomas Henry Huxley, 1880
MARSUPIALIA Illiger, 1811
*SINBADELPHYS SCHMIDTI* Cifelli, 2004

**Figure 14:**
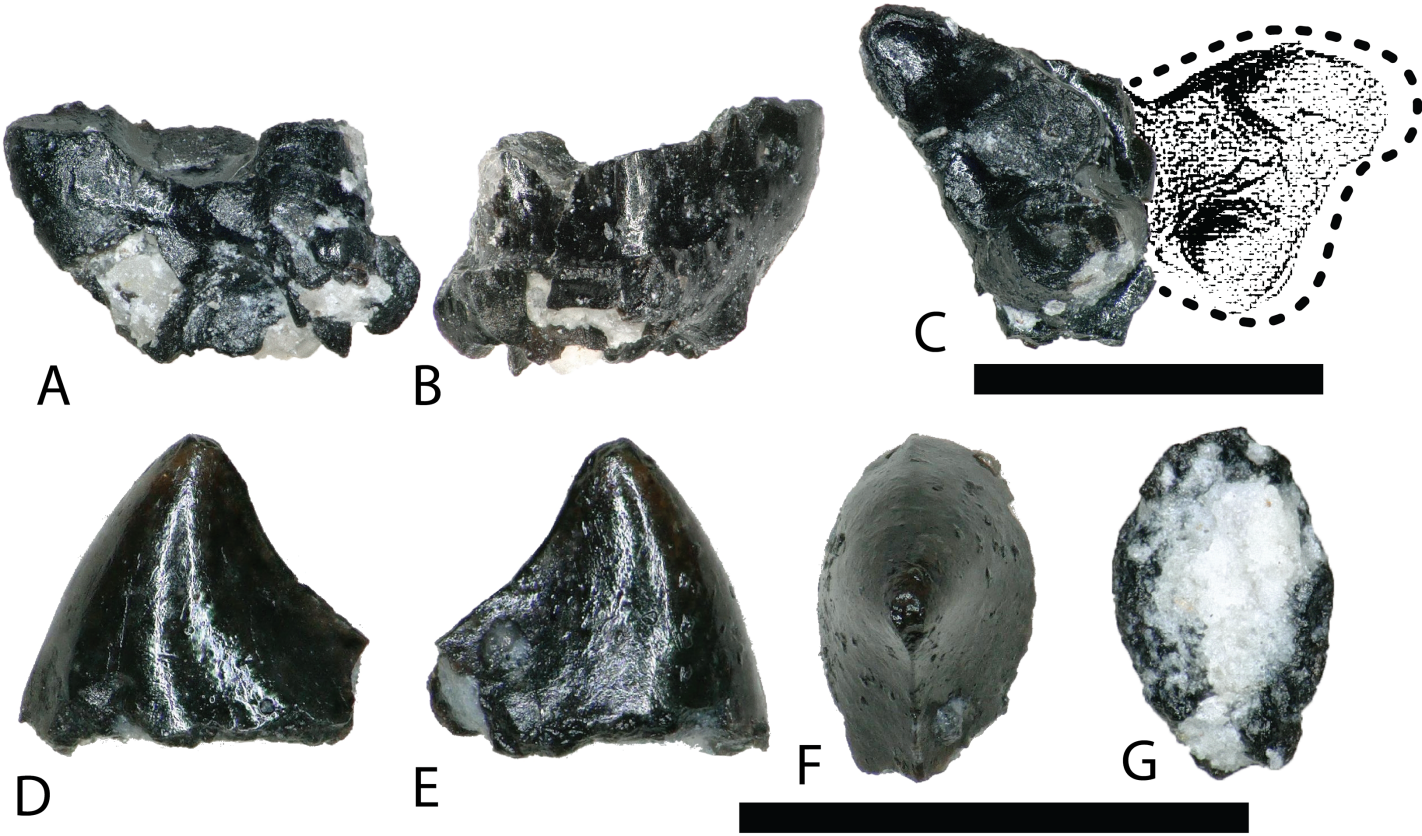
Mammalian teeth. (A–C) (NCSM 33354) mesial half of a right M2 upper molar belonging to a small marsupial, in (A) lingual view (showing plane of fracture), (B) labial view, and (C) occlusal view. Dotted line and shaded area represent estimated dimensions of complete tooth, based on complete upper left M2 molar of *Sinbadelphys schmidti* (Cifelli, 2004). (D–G) (NCSM 33355) marsupial upper premolar in (D–E) labial?/lingual?, (F) occlusal, and (G) basal views. Scale bars equal one millimeter

NCSM 33354 (Figs. 14A–14C) appears to be the mesial half of a right upper molar at the second locus, possibly belonging to *Sinbadelphys schmidti* but also similar to *Adelodelphys muizoni* (Cifelli, 2004). NCSM 33354 lacks a number of dental features except for the ectoflexus, post-metacrista, and metacone. This makes its precise taxonomic assignment on the basis of dental features difficult. Nonetheless, the dimensions of NCSM 33354 compare favorably to those of *S. schmidti*. The second molar of *S. schmidti* (OMNH 26451) has an anteroposterior length of 1.5 mm, and another possible second molar (OMNH 33088) has an anteroposterior length of 1.89 mm. NCSM 33354 has a predicted anteroposterior length ranging from 1.5 to 1.6 mm. Therefore, *S. schmidti* is the most appropriate known taxon to ascribe to NCSM 33354.

MARSUPIALIA indet.

NCSM 33355 (Figs. 14D–14G) is the incomplete upper, second premolar of a marsupial, most likely belonging to *A. muizoni*. This assignment to a marsupial clade is based on comparisons with illustrations in Lillegraven (1969) and Clemens (1966). The dimensional proportions of NCSM 33355 compare favorably with Cretaceous marsupial teeth at the second locus, and the height of the principal cusp indicates it is a maxillary tooth. The length of the second premolar of Cretaceous marsupials is approximately 80% the length of the first lower molar (Lillegraven, 1969; Cifelli, 2004). If NCSM 33355 was complete it would be approximately 1.1 mm in length, 0.2 mm smaller than the estimated length of the M1 of *A. muizoni* (1.3 mm). Therefore, NCSM 33355 is mostly likely referable to *A. muizoni* although the specimen could also belong to the slightly larger *S. schmidti,* which has lower molars lengths between 1.4 and 1.7 mm and estimated premolar lengths between 1.2 and 1.4 mm.

Ichnofossils
ELONGATOOLITHIDAE Zhao, 1975
*MACROELONGATOOLITHUS* Li, Yin, and Liu, 1995
*MACROELONGATOOLITHUS* SP.

**Figure 15:**
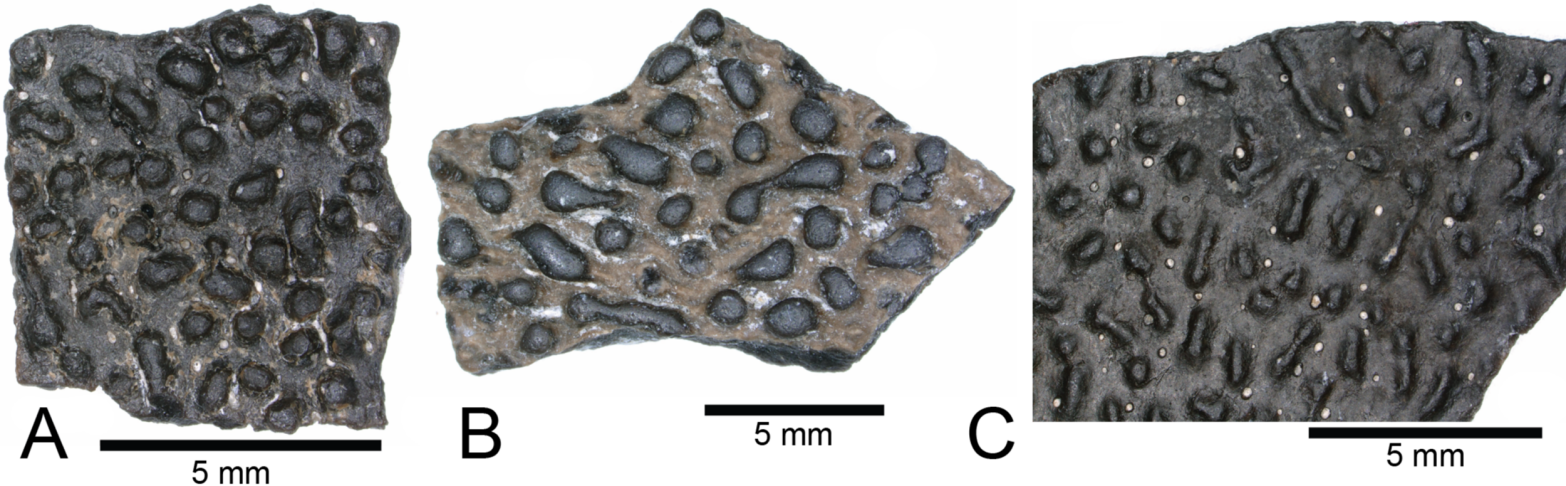
*Macroelongatoolithus* egg shell. (A–C) (NCSM 33364, NCSM 33363, NCSM 33365) *Macroelongatoolithus* egg shell fragments. Scale bars equal five millimeters.

A total of 28 fragments of eggshell, belonging to the oogenus *Macroelongatoolithus*, were recovered from the COI. These fragments exhibit varying degrees of wear and range between 6–20 mm^2^ and 1–3 mm thick (Figs. 15A–15C). Fragments usually possess a subtle curvature along a single axis, suggesting an elongated egg morphology (Zelenitsky, Carpenter & Currie, 2000). Shell material displays variation in ornamentation, similar to material previously collected in the Mussentuchit Member (Zelenitsky, Carpenter & Currie, 2000). These include dispersituberculate and linearituberculate ornamentation, with some fragments having well-defined, bulbous nodes and tubercles.

### Biodiversity and paleoecology

**Figure 16:**
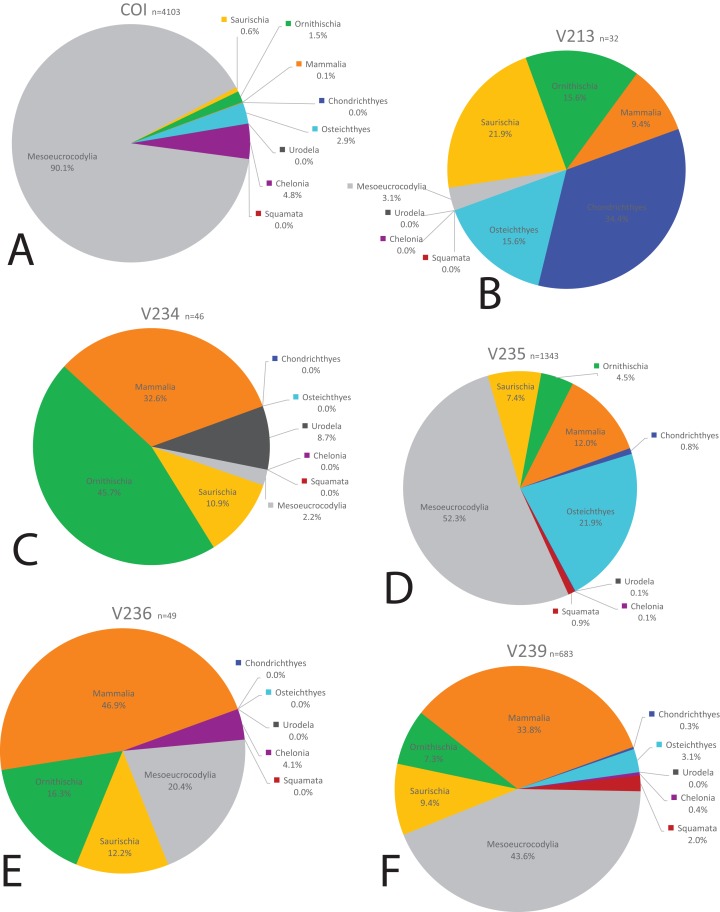
Biodiversity pie charts of the COI and five OMNH microfossil localities. Pie charts representing the biodiversity from (A) COI, (B) OMNH V213, (C) OMNH V234, (D) OMNH V235, (E) OMNH V236, and (F) OMNH V239.

**Figure 17:**
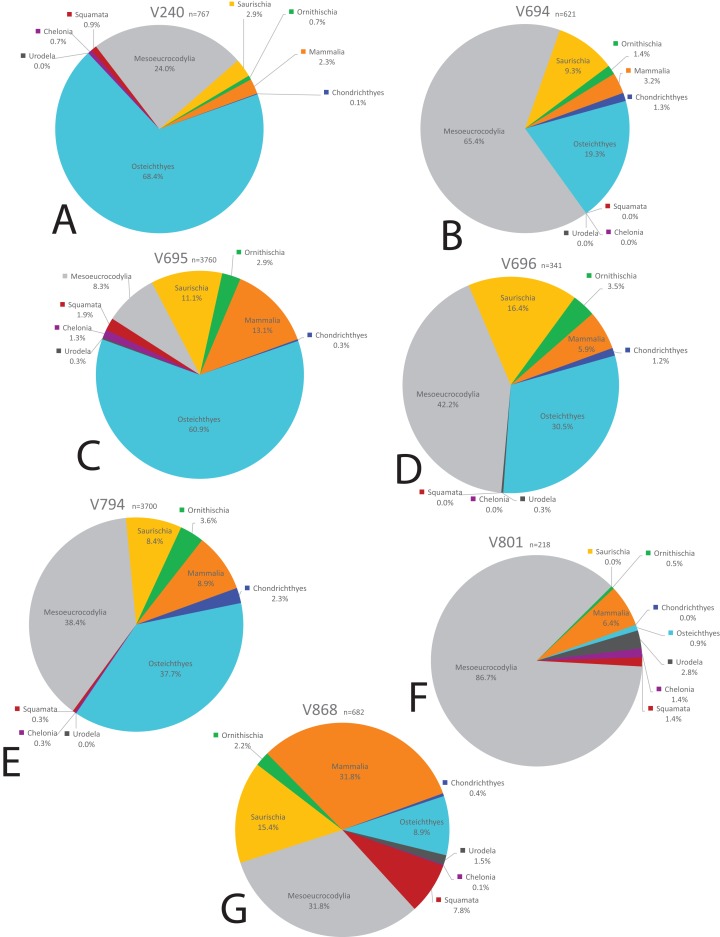
Biodiversity pie charts of seven OMNH microfossil localities. Pie charts representing the biodiversity from (A) OMNH V240, (B) OMNH V694, (C) OMNH V695, (D) OMNH V696, (E) OMNH V794, (F) OMNH V801, and (G) OMNH V868.

We compared the faunal assemblage of the COI sight to 12 OMNH microvertebrate localities from the Mussentuchit Member sourced from Goldberg (2000) (see Data S2 for complete database). Results reveal that the total proportions of all specimens from all sites is skewed toward crocodylomorphs and osteichthyans, constituting 46% and 30%, respectively, whereas mammals are the third most abundant taxonomic group at 10%. Dinosaurian material as a whole makes up 10%, but when the two largest clades are separated their abundance proportion drops (Saurischia at 7% and Ornithischia at 3%). Squamata, Chondrichthyes, Chelonia, and Urodela have the lowest abundances, together making up the remaining 4% (Figs. 16 and 17).

Eight of the 13 Mussentuchit Member sites analyzed are dominated by mesoeucrocodylian remains. This pattern is also present at a site not included in this study (McDonald et al., 2017), raising that value to nine of 14 sites. The most common taxon of secondary abundance in mesoeucrocodylian-dominated localities are osteichthyan fish, although this proportion is nearly equal with sites containing a secondary abundance of mammalian remains (Goldberg, 2000; McDonald et al., 2017). A large percentage of sites are dominated by fish fossils, principally osteichthyans, with secondary abundances represented by mammals, mesoeucrocodylians, and saurischian dinosaurs.

**Figure 18:**
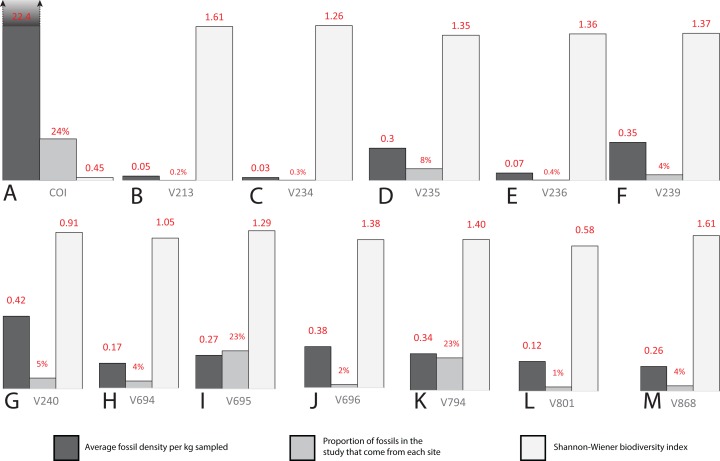
Biodiversity bar charts of the COI and 12 OMNH microfossil localities. Barcharts representing the biodiversity from (A) COI, (B) OMNH V213, (C) OMNH V234, (D) OMNH V235, (E) OMNH V236, (F) OMNH V239, (G) OMNH V240, (H) OMNH V694, (I) OMNH V695, (J) OMNH V696, (K) OMNH V794, (L) OMNH V801, and (M) OMNH V868. The dark gray line represents the density of fossils from a single kilogram of sediment at each locality, the medium gray line shows the proportion of fossils in this study that come from each site, and the light gray line is the Shannon–Wiener biodiversity index for each site.

Three sites (COI, OMNH V695, and OMNH V794) account for the majority of fossil material, each contributing nearly a quarter of the total specimens (Fig. 18). Whereas the COI contributes the most specimens to the dataset, it is the least sampled site, with only 183 kg of sediment processed. This gives the COI a fossil density of 22.4 fossils per kg, 53.33% higher than the next densest site (OMNH V240) with a fossil density of 0.42 fossils per kg (Table 1; Fig. 18).

**Table 1:** Microfossil locality compilation.

	COI	OMNH V213	OMNH V234	OMNH V235	OMNH V236	OMNH V239	OMNH V240	OMNH V694	OMNH V695	OMNH V696	OMNH V794	OMNH V801	OMNH V868	Total
Total specimens	4,100	37	46	1,413	64	695	808	632	3,979	364	3,877	218	710	16,943
Matrix (kg)	183	775	1,825	4,690	910	1,960	1,915	3,645	14,580	955	11,390	1,820	2,780	47,428
Fossil density	22.4	0.05	0.03	0.3	0.07	0.35	0.42	0.17	0.27	0.38	0.34	0.12	0.26	
Proportion	0.24	0.002	0.003	0.08	0.004	0.04	0.05	0.04	0.23	0.02	0.23	0.01	0.04	
S–W index	0.45	1.61	1.26	1.35	1.36	1.37	0.91	1.05	1.29	1.38	1.40	0.58	1.61	

**Note:**

Specimen abundances, quantity of processed matrix, fossil density, and biodiversity indexes from all 13 localities.

**Figure 19:**
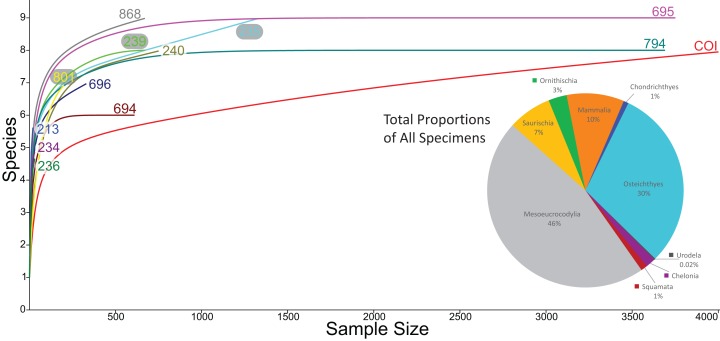
Rarefaction curves for all microfossil localities and biodiversity pie chart of all 13 localities combined. Rarefaction curves for the 12 OMNH microfossil localities listed in the Goldberg (2000) dataset and the Cliffs of Insanity locality. Colors of site names correspond to the rarefaction line of the same color. The pie chart represents the combined percentage of all 13 site abundances used in the present study.

Rarefaction of the sampled faunas across all localities (Fig. 19) shows that only four of the 13 localities (OMNH 239, 694, 695, 794) have plateaued. In other words, these sites have been sampled enough that it is unlikely a new vertebrate clade will be discovered with increased sampling. The COI site does not appear close to leveling off; therefore, a high probability exists to discover new clades not currently documented in the site fauna from further sampling. After estimating the rarefaction over 4,000 specimens, COI appears equal to OMNH V695 in clade representation. The direction of the rarefaction line for the COI locality is quite different from the OMNH localities, rising at a gradual pace to the end of the graph. This behavior is due to the high number of mesoeucrocodylian teeth and osteichthyan fossils in our sample along with the rarer individual numbers of additional clades. In short, the rarefaction of the COI database continuously provides excessive individuals of mesoeucrocodylians and fish along with occasional other clades, which when amalgamated across the entire resampling protocol produces the slowly increasing line. Compared to other OMNH localities there are key clades that are missing from the COI site, and recalculation of rarefaction curves at lower taxonomic levels might have different outcomes than presented here, yet more refined taxonomic classifications are not yet possible. Given the higher potential for COI to yield additional clades through further sampling, the COI locality could be one of the most taxonomically rich microsites yet described from the Mussentuchit Member.

**Figure 20:**
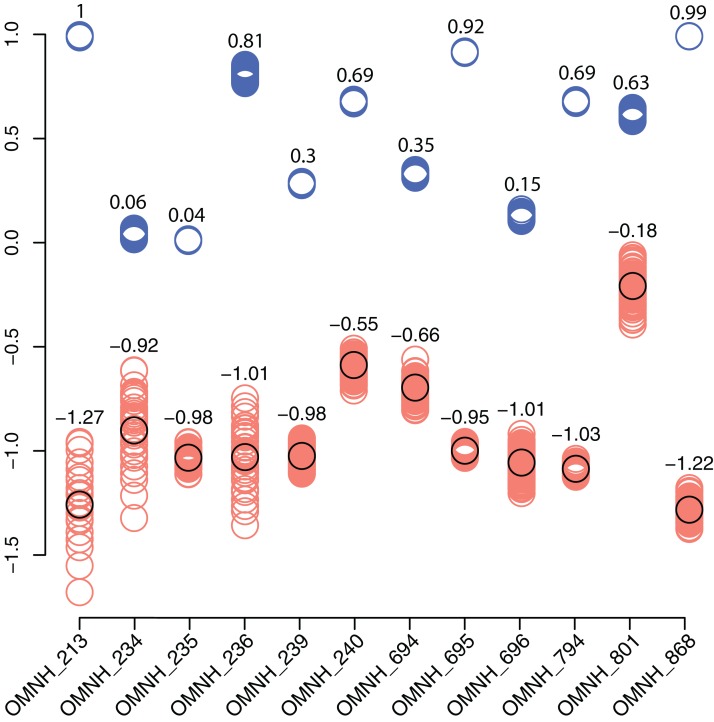
Comparison of subsampled COI Shannon–Wiener indices and Morisita–Horn similarity values compared to OMNH localities. Shannon–Wiener biodiversity index differences between the COI locality and each OMNH locality are shown as orange circles. The black circles show the original Shannon–Wiener value for the COI deposit after subtraction of each OMNH locality. This circle is also the average for the subsampled differences. Morisita–Horn similarity values are shown as blue circles. Each column in the figure contains 100 circles, above which are the averages for each column.

In contrast, the biodiversity as calculated via the Shannon–Wiener index produces a low value, (0.41; Table 1) compared to other OMNH sites. The Shannon–Wiener index takes into consideration both the number of clades present and the number of individuals per clade in order to calculate biodiversity among sites. Sampling localities that have a larger number of clades and a relatively equal number of individuals per clade will receive a higher biodiversity score than sites that have either low number of clades or those sites that possess a large number of clades, yet a very few of those clades possess nearly all of the individual abundance. Therefore, the high number of crocodylomorph fossils from COI compared to all other clades in that site creates an uneven assemblage that effectively lowers this biodiversity measure. Locality OMNH V868 is the most diverse site by this measure, with a biodiversity index of 1.61. Figure 20 shows the results of the Shannon–Wiener COI subsampling. There is an obvious spread of biodiversity values for the COI locality under all subsampling simulations. However, the most distinguishing pattern is that in all cases the biodiversity of this site remains lower than every OMNH locality.

Interestingly, there is no correlation between the number of fossils collected and the Shannon–Wiener biodiversity index. The slope of the correlation was essentially zero (−5.77 × 10^−5^). The four sites that had plateaued rarefaction curves (OMNH V239, V694, V695, and V794) ranged in biodiversity from 1.05 to 1.40, which were all below the peak biodiversity of 1.61 (OMNH V868). This indicates that neither extensive sampling nor abundance of fossils can adequately predict the biodiversity of an individual site.

Similarity, as calculated by the Morisita–Horn index, of the subsampled sites has a much smaller distribution than the Shannon–Wiener values in every comparison, meaning that despite wide differences in biodiversity among subsamples, the similarity of faunas is not sensitive to subsampling techniques. In this index a value of 1 means that all of the clades are shared between localities and the proportions of those clades are also essentially equivalent. Sites OMNH V213 and OMNH V868 have a near identical array of clades compared to COI. The site that is least similar to COI is OMNH V696.

### Paleoenvironmental influences

Goldberg (2000) characterized 11 of the 12 OMNH sites as either fluvial oxbow, floodplain, crevasse splay, or channel deposits, basing these inferences on a number of sedimentary features such as clast size, lag deposits, clay balls, slickensides, and plant debris. Among these localities, OMNH V236 and OMNH V239 were interpreted as channel deposits, OMNH V235 and OMNH V240 as splay environments, OMNH V694 and OMNH V695 as floodplain deposits, and OMNH V801 as an abandoned oxbow channel (Fig. 1D). Some uncertainty was noted for select sites including OMNH V794 and OMNH V868, which were interpreted as either channel deposits or splays, and OMNH V234, which was interpreted as either a floodplain or splay deposit.

**Figure 21:**
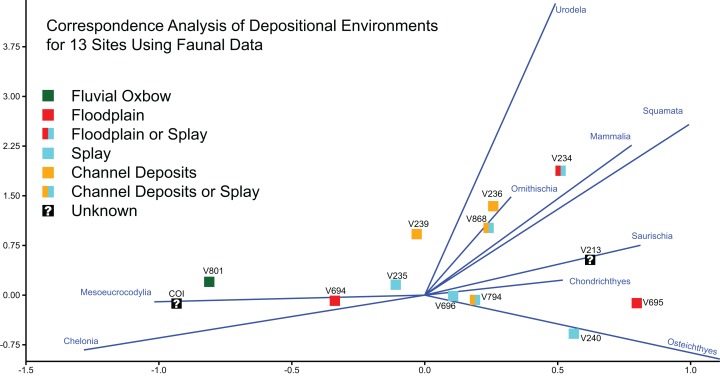
Correspondence analysis of depositional environments for 13 sites using faunal data. Correspondence analysis of all 13 sites, using simplified faunal data as a predictor of depositional environment. Blue eigenvectors represented by faunal groups.

Correspondence analysis of all 13 sites, using faunal data as a predictor of depositional environment (Fig. 21), shows that site faunal compositions can be associated with specific depositional environments; this ordination plots the COI close to OMNH V801, both of which are interpreted as representing oxbow lake environments. These sites trend to the bottom left of the ordination space due to their high concentration of Mesoeucrocodylia and Chelonia material. However, for the majority of sites taxonomic content is a poor predictor of depositional environment. These include channel or splay deposits and floodplain deposits.

### Stratigraphic and geographic context

Goldberg (2000) noted that determining the exact stratigraphic position of the OMNH localities in the Mussentuchit Member is challenged by difficult terrain, distances between sites, and frequently obscured bedding. Nonetheless, Cifelli et al. (1999) and Goldberg (2000) were able to place ten of the microsites in relative positions to one another both stratigraphically (Fig. 1D) and geographically (Fig. 1B). The relative stratigraphic positions were based on the proximity of a site to the upper or lower contacts of the Mussentuchit Member, or its position above or below a specific ash layer used as a marker bed. To this framework, we added the stratigraphic position of the COI as determined from the contact with the Naturita Formation.

The COI and OMNH V239 are the stratigraphically highest units, located in the uppermost strata of the Mussentuchit Member. OMNH V235, OMNH V694, and OMNH V794 are located above the ash layer, whereas OMNH V868 and OMNH V695 are located directly below the ash layer. OMNH V240, OMNH V801, and OMNH V234 are all located near the bottom of the Mussentuchit Member, just above the contact with the Ruby Ranch Member. Geographically, OMNH V801 represents the Northeastern-most locality, while the COI represents the Southwestern-most.

## Discussion

### Biodiversity

Prior studies of microvertebrate localities in the Mussentuchit Member of the Cedar Mountain Formation reportedly processed 49,295 kg of matrix in total, with individual sites contributing between 775 and 14,580 kg of matrix. These studies have resulted in a total of 13,036 identifiable specimens (Goldberg, 2000). By comparison, the COI microvertebrate locality preserves a uniquely high density of fossils, with over 6,342 specimens recovered from only 183 kg of sediment.

Baenid, helochelydrid (solemydid), glyptopsid, and trionychid turtles have been reported previously (Cifelli et al., 1997; Fiorillo, 1999; Herzog, Zanno & Makovicky, 2015) from the Mussentuchit Member. Our histological analysis as well as qualitative assessment of turtle shell fragments from the COI add the presence of Adocidae, representing the first occurrence of this taxon from the Cedar Mountain Formation and the earliest occurrence in North America.

The majority of Mussentuchit Member sites sampled, including the COI locality, are dominated by mesoeucrocodylian teeth (Goldberg, 2000; McDonald et al., 2017), with a large percentage of sites alternatively dominated by fish materials (osteichthyans and chondrichthyans). The remarkably high abundance of mesoeucrocodylians (90% of recovered fossils) warrants further investigation as a potential paleoenvironmental signal within the Mussentuchit Member itself. We hypothesize that the general predominance of mesoeucrocodylians in the upper Cedar Mountain Formation likely represents an authentic paleoecological signal tied to transgression of the WIS during this interval. If specimen NCSM 33308 is indeed an enchodontid tooth, then this would support our hypothesis because members of Enchodontidae are predominantly marine (Silva & Gallo, 2011). However, shed teeth can often over-represent a population density, and the fluvial nature of many of the sampled localities, (including the COI) can lead to the overrepresentation of aquatic faunal elements compared to terrestrial vertebrate elements.

A diverse assemblage of dinosaurian species are known from the Mussentuchit Member based on previous microfaunal studies (Eaton, 1987; Nelson & Crooks, 1987; Cifelli et al., 1999; Fiorillo, 1999; Kirkland et al., 1999). We find support for some of these identifications in the COI sample; however, we take a more conservative approach to isolated tooth identifications based on our knowledge of the unique taxonomic diversity of Mussentuchit Member macrofaunal remains (i.e., generally an undescribed macrofauna consisting of new dinosaurian species; Makovicky, Zanno & Gates, 2015; Zanno & Makovicky, 2016) and previous studies cautioning on the assignment of isolated dinosaur teeth to refined taxonomic levels (Zanno et al., 2013). Importantly, we provide some of the first detailed descriptions and photographic documentation of dinosaurian teeth from the Mussentuchit Member.

Our gross morphological examination of isolated theropod teeth suggests the presence of most major dentigerous coelurosaurian clades known to have inhabited the Western Interior Basin of North America during the Late Cretaceous, including Tyrannosauroidea, Dromaeosauridae, and Avialae, which is consistent with the diversity reported by previous workers (Eaton, 1987; Nelson & Crooks, 1987; Cifelli et al., 1999; Fiorillo, 1999; Kirkland et al., 1999). Tyrannosauroidea is definitively represented by the presence of a single premaxillary tooth similar in morphology to tyrannosauroid premaxillary teeth from the Early Cretaceous of Asia and North America (Zanno & Makovicky, 2011).

However, it remains difficult to parse out more refined taxonomic identifications for many theropod teeth due to the lack of associated skeletal material, and some tooth diversity may be explained by heterodonty and variation along the tooth row (Smith, 2002, 2005; Buckley, 2009). Furthermore, it is unclear the degree to which ontogeny contributes to dental variation among theropods. Ontogeny is noted to have only minor effects on the dental shape of *Coelophysis bauri* teeth, specifically the degree of tooth recurvature; however, variation is noted in the presence of discrete tooth traits such as denticles on premaxillary teeth or longitudinal ridges on tooth crowns (Buckley, 2009), which can affect identifications.

Several morphotypes of ornithischian teeth are represented at COI, suggesting a large diversity of herbivorous dinosaurs, most of which are already documented via published or undescribed macrovertebrate remains (Kirkland, 1998; Carpenter et al., 1999; Makovicky, Zanno & Gates, 2015; Zanno & Makovicky, 2016). A quantitative morphological analysis coupled with publication of these specimens will aid in producing more refined identifications.

Marsupial mammals are abundant in Mussentuchit Member localities generally (Goldberg, 2000), yet make up only a minor component of the COI diversity. The COI site thus far lacks chondrichthyan or serpent remains; yet, rarefaction curves (Fig. 19) suggest the likelihood of recovering materials representative of these clades with additional sampling efforts.

Biodiversity and similarity analyses of microvertebrate localities from the Mussentuchit Member indicate substantial variation in both the types of animals and their relative abundances throughout the member. The COI locality is considerably more fossiliferous as coded in our dataset compared to the OMNH sites; however, it is unclear how pronounced this signal is due to known sampling biases between institutions. Subsampling the former site provides a means to equilibrate the faunas prior to running biodiversity statistics, as well as producing a distribution of possible biodiversity values. Each of the subsamples shows what the difference in biodiversity would be if a subset of the COI fauna was uncovered as opposed to the current large sample size. This exercise shows that depending on the sample obtained from a particular locality, inferences about the biodiversity can be quite disparate, underscoring the need for as much sediment collection as possible. Although more detailed analyses and more extensive field sampling is required to gain a fuller picture, it seems reasonable that changes to paleoenvironment through the transgressing WIS would have a major impact on the species that could live in the Mussentuchit Member depositional basin. Compounding this potential faunal change are alterations to the depositional regime as sea level rose, playing a role in burying the vertebrate microfossil localities.

### Paleoenvironmental and taphonomic considerations

Taphonomic processes such as transport, sorting, winnowing, reworking, time averaging, and other hydrodynamic factors have been suggested to affect the comparability between sites and perhaps introduce significant bias (Wilson, 2008). However, Rogers et al. (2017), compared six intraformational vertebrate microfossil bonebeds in the Upper Cretaceous Judith River Formation, represented by three lacustrine settings and three channel-hosted settings, and found them to be largely comparable with regards to taphonomic bias.

We found little evidence among currently sampled microvertebrate localities in the Mussentuchit Member to support a relationship between depositional environment (Fig. 21) or stratigraphic position (Fig. 1D) and faunal abundance data, with the exception of the two sites proposed to be oxbow lake environments (COI and OMNH V801). Although ambiguity in interpretations of depositional environment for some OMNH localities (e.g., V868, 794) (Goldberg, 2000), if clarified, could extend this result to channel deposits as well.

The faunal composition of the COI plots closest to OMNH V801 in our correspondence analyses; both localities are characterized by a relatively high concentration of turtle and mesoeucrocodylian material. This raises the possibility that relative overabundance of semiaquatic taxa within these sites represents a true paleoenvironmental signal, yet we note that a sample size of two is insufficient to generate much confidence. Moreover, significant differences remain between these two localities suggesting that they may be non-isotaphonomic. OMNH V801 preserves an abundance of articulated fossils, including complete turtle shells, and freshwater invertebrates with mottled coloration, a feature proposed to indicate high biological activity (Goldberg, 2000). In contrast, we have not recovered freshwater invertebrates or articulated fossils from the COI locality, and turtle shell material is fragmentary. These dissimilarities suggest that, although depositional environment may be broadly influencing the proportion of vertebrate clades preserved at these localities, different taphonomic factors are also at work and the sites may not be taphonomically equivalent.

### Paleobiogeography

The late Early Cretaceous Laurasian Interchange Event, EKLInE, is marked by a prolonged interval of first occurrences of taxa bearing Asian affinities in North America during the Aptian–Cenomanian (Cifelli et al., 1999; Zanno & Makovicky, 2011, 2013) including hadrosauroids (Norman, 2004; McDonald, Wolfe & Kirkland, 2010b), pachycephalosaurians (Cifelli et al., 1997; Gangloff, 1998; Garrison et al., 2007), neoceratopsians (Farke et al., 2014), and theropods (e.g., tyrannosauroids; Zanno & Makovicky, 2011, ornithomimids; Longrich, 2008, and oviraptorosaurs; Makovicky & Sues, 1998), mammals (Cuenca-Bescós & Canudo, 2003), squamates (Nydam, 2002; Nydam, Rowe & Cifelli, 2013), and trionychid turtles (Hirayama, Brinkman & Danilov, 2000) and is considered one of the major factors leading to the extinction and replacement of much of North America’s endemic dinosaur clades (Kirkland et al., 1997, 1999).

The previous oldest occurrence of adocid turtles in North America derives from the Smoky Hollow Member of the Straight Cliffs Formation (Eaton et al., 1999), dated to the Turonian (∼92 Ma; Titus & Loewen, 2013). Therefore, the presence of adocids in the Cenomanian aged (∼97 Ma) Mussentuchit Member (Cifelli et al., 1997; Garrison et al., 2007) extends the presence of the clade in North America by approximately 5 million years and provides further evidence for Laurasian interchange between Asia and North America at or prior to the dawn of the Late Cretaceous.

The oldest records of adocids derive from the Late Jurassic of Asia (Syromyatnikova & Danilov, 2009; Danilov, Sukhanov & Syromyatnikova, 2011), and the clade is notably absent in the fossil record of Europe during the Early Cretaceous, suggesting a Beringian dispersal route in concordance with the majority of other vertebrate records. It has been suggested that the high-latitude connection between eastern Asia and western North America acted as a climatic barrier, resulting in a dispersal filter that restricted smaller members of faunal assemblages such as turtles (Lipka et al., 2006). The appearance of adocids in North America by at least the Cenomanian, would seem to contradict this hypothesis, suggesting that at least some testudinatans were able to disperse across high latitude barriers in the Early Cretaceous. This is especially notable given that turtles have been suggested to exhibit a high degree of endemism and climatic zonation (Moody, 1984; Gates et al., 2010; Hirayama, Brinkman & Danilov, 2000); however, extant turtles endemic to higher latitudes display broader geographic ranges compared to those at lower latitudes (Angielczyk, Burroughs & Feldman, 2015).

## Conclusions

The recovery and analyses of over 4,000 new fossil specimens from the COI microvertebrate site has improved our understanding of the Cenomanian vertebrate fauna from Mussentuchit Member of the Cedar Mountain Formation. The site is diverse and includes the remains of Amiidae, lepisosteiforms, pycnodontiforms, salmoniforms, albanerpetontids, helochelydrids, adocids, mesoeucrocodylians, tyrannosauroids, dromaeosaurids, the theropods *Richardoestesia* and *Paronychodon*, avialans, a variety of ornithischians, metatherians, and trace fossils assigned to *Macroelongatoolithus*. The recovery of adocid turtle remains marks a new addition to the Mussentuchit Member assemblage and adds to a growing body of evidence refining the pattern and tempo of faunal interchange between Asia and North America prior to and post the Early/Late Cretaceous boundary. It also represents the earliest occurrence of Adocidae in North America, extending the record of this group by 5 million years. Documenting the fine-scale patterns of the Mussentuchit Member taxa can provide a better understanding of the effects of biogeographic complexity during the Cretaceous of North America.

Our morphometric tests of the eight theropod teeth from the COI using the Hendrickx, Mateus & Araújo (2014) database were informative, yet limited in their ability to accurately predict taxonomic associations. The inconsistencies between where a tooth may plot is likely due to the inability of existing databases to account for subtle shape variation and ambiguous taxonomic referrals due to their reliance solely on a limited series of linear measurements. Future attempts to refine this methodology will incorporate a larger sample set of theropod teeth from the Mussentuchit Member utilizing landmark data and three-dimensional shape analyses.

Finally, we find no consistent relationship between depositional environment and the abundance of vertebrate clades between microvertebrate localities in the Mussentuchit Member (with the possible exception of oxbow environments, which may preserve an overabundance of semiaquatic taxa). Yet also caution that detailed taphonomic studies are needed to parse out whether the lack of paleoenvironmental signal for most depositional environments is the result of non-isotaphonomy or is authentic.

## Supplemental Information

10.7717/peerj.5883/supp-1 Supplemental Information 1 Theropod tooth linear measurement and ratio data. Linear measurements and tooth measurement ratios of theropod teeth from the COI combined with the Hendrickx and colleagues (2014) database and the merged and modified Larson & Currie (2013) and Williamson & Brusatte (2014) databases.

10.7717/peerj.5883/supp-2 Supplemental Information 2 Biodiversity data of microfossil localities. Biodiversity data for the COI and all 12 OMNH microfossil localities.

10.7717/peerj.5883/supp-3 Supplemental Information 3 Cliffs of Insanity NCSM catalogue numbers and corresponding temporary names. Total taxonomic fossil counts for the COI, with corresponding NCSM catalogue numbers and other collection data.

10.7717/peerj.5883/supp-4 Supplemental Information 4 Principal component and linear discriminant analyses. PCAs and LDAs of the eight COI theropod teeth combined with a merged and modified Larson & Currie (2013) and Williamson & Brusatte (2014) database.

10.7717/peerj.5883/supp-5 Supplemental Information 5 R file containing the results of the Morisita-Horn and Shannon-Wiener subsampling trials.

10.7717/peerj.5883/supp-6 Supplemental Information 6 R file containing the subsampled faunas from the COI locality for each calculation. This file is an R list where each list element is a difference OMNH locality comparison. There are 100 columns each corresponding to a different subsampling trial. Rows are the fauna from our entire dataset.

10.7717/peerj.5883/supp-7 Supplemental Information 7 R code used in this study.

10.7717/peerj.5883/supp-8 Supplemental Information 8 Subsampling results as a CSV file. Rows are each of the 100 comparisons and columns are the OMNH localities that the COI locality is compared against.

## Institutional Abbreviations

NCSM: North Carolina Museum of Natural Sciences
OMNH: Sam Noble Museum of Natural History, University of Oklahoma.

## Anatomical Abbreviations and Terminology

ADM: anterior denticles per millimeter
AL: apical length
BW: basal width
CBL: crown basal length
CBR: crown base ratio
CBW: crown basal width
CH: tooth crown height
CHR: crown height ratio
DC: distocentral denticle density
FABL: fore aft basal length
MC: mesiocentral denticle density
MCL: mid-crown length
MCR: mid-crown ratio
MCW: mid-crown width
PDM: posterior denticles per millimeter Anatomical terminology follows Hendrickx, Mateus & Araújo (2015).

## Additional Information and Declarations

### Field Study Permissions

The following information was supplied relating to field study approvals (i.e., approving body and any reference numbers): COI materials were collected under State of Utah permit (#2014-447).

### Data Availability

The following information was supplied regarding data availability: The raw data and code are provided in the Supplemental Files.
