# Supplementary material for: A new microvertebrate assemblage from the Mussentuchit Member, Cedar Mountain Formation: insights into the paleobiodiversity and paleobiogeography of early Late Cretaceous ecosystems in western North America
Source: PeerJ. 2018 Nov 16;6:e5883. doi: 10.7717/peerj.5883 (PMC6241397; doi:10.7717/peerj.5883)
Supplement: Supplemental Information 4 — PCAs and LDAs of the eight COI theropod teeth combined with a merged and modified Larson & Currie (2013) and Williamson & Brusatte (2014) database. [file peerj-06-5883-s004.pdf]

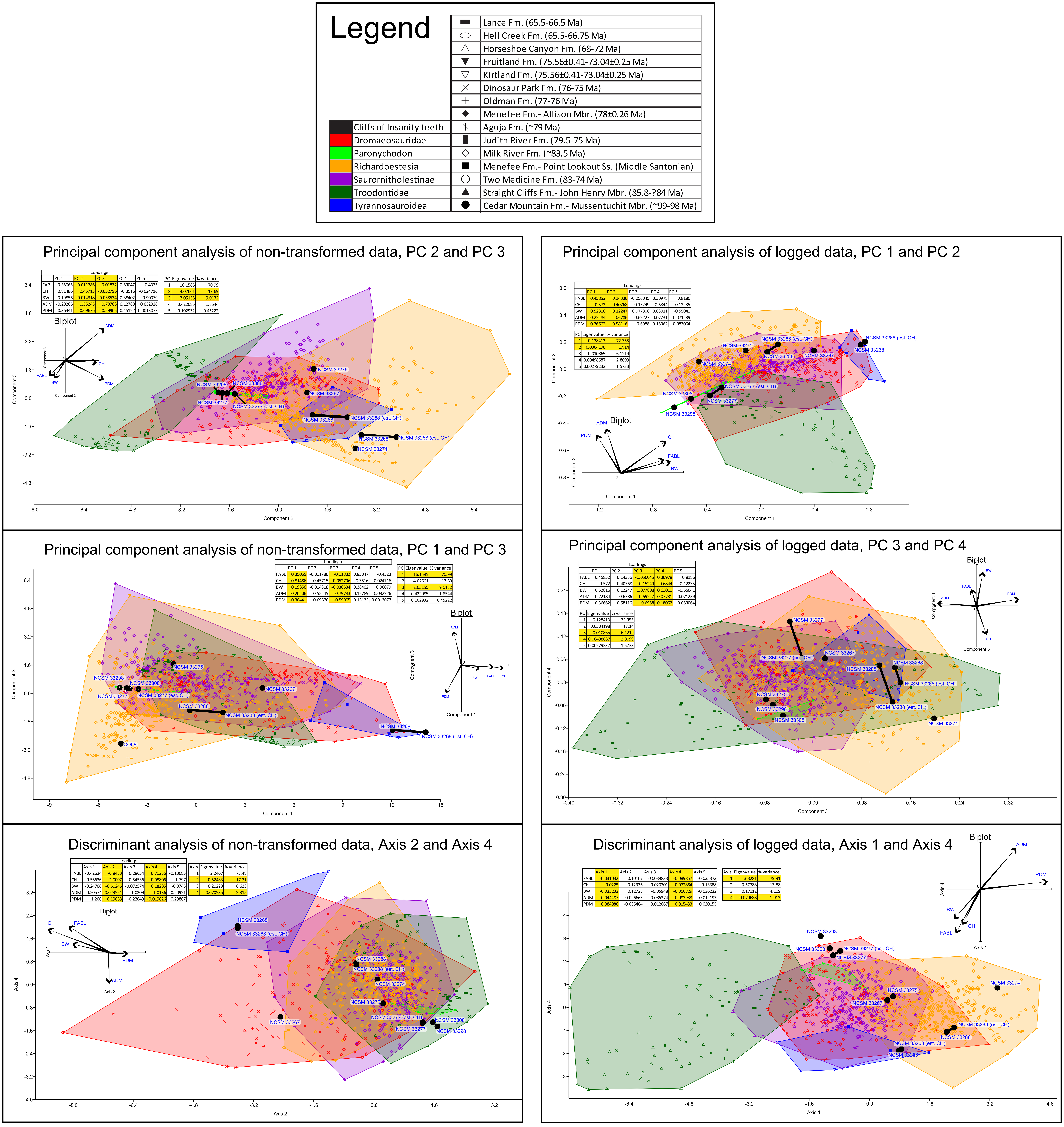

Legend
Lance Fm. (65.5-66.5 Ma)
Hell Creek Fm. (65.5-66.75 Ma)
Horseshoe Canyon Fm. (68-72 Ma)
Fruitland Fm. (75.56±0.41-73.04±0.25 Ma)
Kirtland Fm. (75.56±0.41-73.04±0.25 Ma)
Dinosaur Park Fm. (76-75 Ma)
Oldman Fm. (77-76 Ma)
Menefee Fm.- Allison Mbr. (78±0.26 Ma)
Aguja Fm. (~79 Ma)
Judith River Fm. (79.5-75 Ma)
Milk River Fm. (~83.5 Ma)
Menefee Fm.- Point Lookout Ss. (Middle Santonian)
Two Medicine Fm. (83-74 Ma)
Straight Cliffs Fm.- John Henry Mbr. (85.8-?84 Ma)
Cedar Mountain Fm.- Mussentuchit Mbr. (~99-98 Ma)
Cliffs of Insanity teeth
Dromaeosauridae
Paronychodon
Richardoestesia
Saurornitholestinae
Troodontidae
Tyrannosauroidea
Principal component analysis of non-transformed data, PC 2 and PC 3
Principal component analysis of logged data, PC 1 and PC 2
Principal component analysis of non-transformed data, PC 1 and PC 3
Principal component analysis of logged data, PC 3 and PC 4
Discriminant analysis of non-transformed data, Axis 2 and Axis 4
Discriminant analysis of logged data, Axis 1 and Axis 4
